# Photo-redox catalyzed dehydrazinative acylation of N-heterocycles *via* Minisci reaction†

**DOI:** 10.1039/d1ra07063k

**Published:** 2021-12-01

**Authors:** Saira Hafeez, Aamer Saeed

**Affiliations:** Department of Chemistry, University of Science and Technology of China Hefei Anhui 230026 China; Department of Chemistry, Quaid-I-Azam University Islamabad 45320 Pakistan aamersaeed@yahoo.com asaeed@qau.edu.pk

## Abstract

Visible light-induced acylation of heteroaromatic compounds have been achieved using benzoyl hydrazides as an efficient acyl source under mild reaction conditions. The photo-redox catalyzed oxidative cleavage of hydrazides leads to *in situ* formation of acyl radicals, which subsequently couple with various N-heterocycles to produce acylated products. This synthetic strategy performs the classic Minisci reaction in an eco-friendly and greener way with functional group tolerance and regioselectivity. Control experiments confirm the radical pathway for this transformation.

## Abstract 2

Visible light-induced acylation of heteroaromatic compounds have been achieved using benzoyl hydrazides as an efficient acyl source under mild reaction conditions.

## Graphical abstract

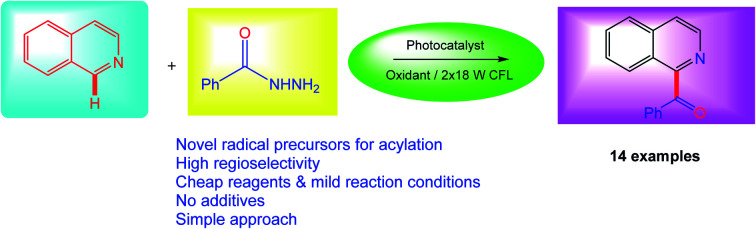

## Introduction

The synthesis of natural products raises the frontiers of synthetic chemistry as it encounters the challenge to construct complex and structurally diverse molecular frameworks.^1–5^ Natural products that show biological activity frequently serve as vital targets for novel drug design assemblies.^6^ Efficient and powerful synthetic strategies are required to access these diverse and structurally complex assemblies, which is a multidimensional challenge for synthetic chemists.^7^ One strategy towards the catalytic activation of organic molecules is visible light photo-redox catalysis.^8^ Visible light photocatalysis has currently received growing attention from organic chemists because of its extensive applications in organic synthesis as well as its consequences for sustainable chemistry.^9^

**Fig. 1:**
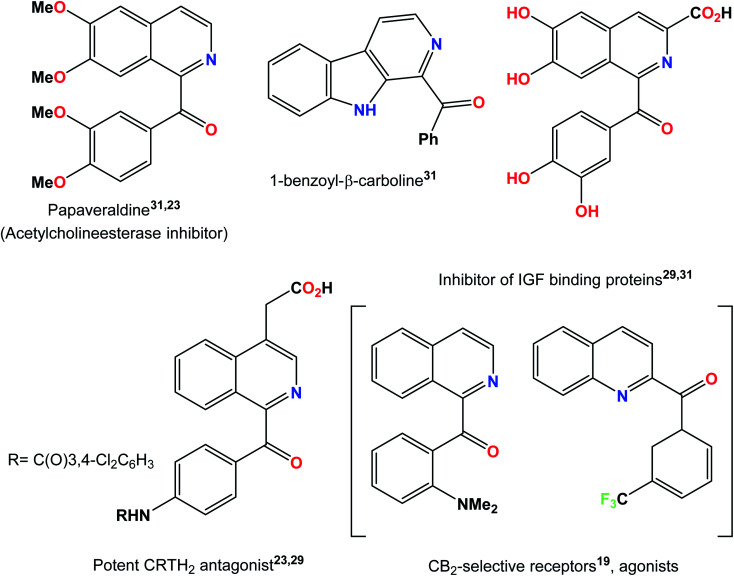
Some acyl functionalized N-heterocycles in natural products and pharmaceutical drugs.

N-heterocycles are key structural units for the synthesis of a variety of natural products with widespread application in the field of medicine. Some representative examples are shown in Fig. 1. For example, benzoylated quinolines possess numerous biological activities like antibacterial, antifungal, and anticancer activities.^10–12^ Different strategies have been designed, of which, Minisci radical C–H functionalization has been considered the most direct and efficient approach to access functionalized N-heterocycles.^13,14^ Numerous metal-free acylation strategies have been reported *via* radical transformations including aldehydes,^15–17^ benzylamines,^18^ aryl methanol derivatives,^19,20^ methyl arenes^21,22^ and α-keto acids^23^ as coupling counterparts along with oxidants at high reaction temperatures. Some milder protocols from previously reported radical precursors have also been reported by Antonchick and Zhang but with the use of hypervalent iodine as an oxidant and an electrochemical approach, respectively.^24,25^ A variety of reactions resulting from visible light mediated approaches have been carried out in order to obtain more mild protocols.^26–28^ In the past few years, the generation of acyl radicals for the acylation of N-heterocycles by the photo-redox strategy have been reported using α-keto acids^29–31^ and terminal alkynes^32^ as the acyl radical source. A visible light-induced catalytic system was also developed recently to achieve the acylation of pyridine *N*-oxides by the decarboxylation of α-oxocarboxylic acids using the organic dye fluorescein dimethylammonium as a new type of photocatalyst.^33^ The philicity of acetonyl and benzoyl radicals has been investigated by R. H. Verschueren and co-workers recently *via* experimental as well as computational studies.^34^

**Fig. 2:**
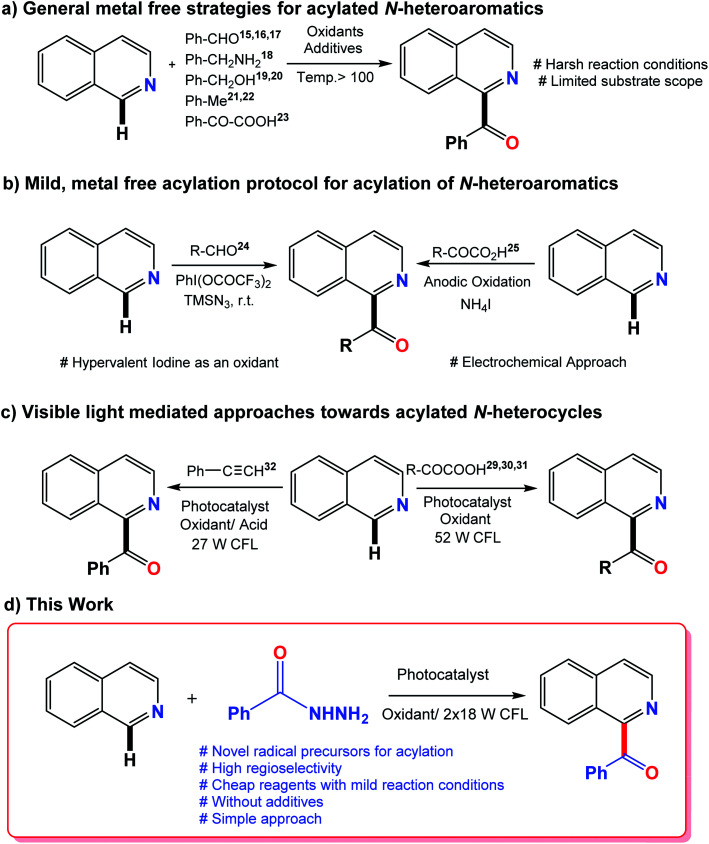
Previous literature reports and the current photo-mediated strategy.

In this context, the production of acyl radicals *via* the oxidative cleavage of hydrazides introduces an outstanding idea. Acyl hydrazides are a good source of acyl radicals in transition metal catalysis^35,36^ but their use in the acylation of N-heterocycles *via* acyl radicals has not been reported yet. In 2017, the oxidative carbamoylation of electron deficient N-heterocycles by hydrazine carboxamide was reported by our group.^37^ Therefore, in continuation of our interest with N-heterocycles, we envisioned the utility of benzoyl hydrazides as an efficient source of acyl radicals under visible light conditions using eosin-Y as a photocatalyst. Recently, eosin-Y has been reported as a direct hydrogen atom transfer photocatalyst to generate acyl radicals from aldehydes as radical precursors.^38^ The generation of acyl radicals takes place very easily by oxidative cleavage using oxidants. Traditional Minisci reactions require the use of harsh conditions such as high temperature excess amounts of radical precursors, long reaction times and poor site selectivity. The current strategy eliminates these requirements with the introduction of novel radical precursors for the acylation of heterocycles. Previously reported methods and the current photo-mediated strategy are given in Fig. 2.

## Results and discussion

To initiate the study of this transformation, a model reaction between benzoyl hydrazine (1) and isoquinoline (2) was carried out under visible light from 18 W CFL bulbs in the presence of a photocatalyst and an oxidant using DMSO/H_2_O (4 : 1) at room temperature. The benzoylated product was obtained with 49% yield using Ru(bpy)_3_Cl_2_·6H_2_O and K_2_S_2_O_8_ after 12 hours (Table 1 entry 2). The influence of various photocatalysts was then investigated which showed results with moderate to good yields (entries 3–5). The best result is obtained using eosin-Y in combination with K_2_S_2_O_8_ (entry 3). Screening studies of different oxidants showed that K_2_S_2_O_8_ gives the highest yield among all others tested (entries 6–9). Increase or decrease in reaction time results in low yield of the product (entries 10 and 12). The optimal solvent system (DMSO/H_2_O 4 : 1) was obtained as all other solvents gave poor conversions (entries 12–18).

**Table 1:** Optimization study of dehydrazinative acylation of isoquinoline^a^

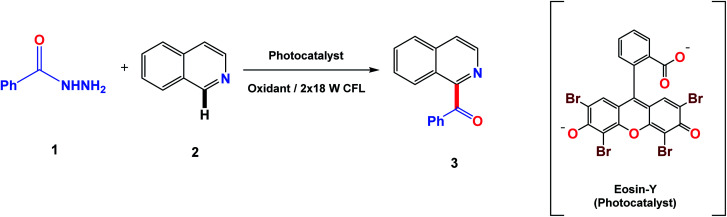					
Entry	Photocatalyst (mol%)	Oxidant (mmol)	Solvent (mL)	Time (h)	Yield^b^ (%)
1^a^	—	K_2_S_2_O_8_	DMSO/H_2_O	12	11
2	Ru(bpy)_3_Cl_2_·6H_2_O	K_2_S_2_O_8_	DMSO/H_2_O	12	49
3	Eosin-Y	K_2_S_2_O_8_	DMSO/H_2_O	12	80
4	9-Fluorenone	K_2_S_2_O_8_	DMSO/H_2_O	12	62
5	Ir-PC1	K_2_S_2_O_8_	DMSO/H_2_O	12	56
6	Eosin-Y	TBHP	DMSO/H_2_O	12	61
7	Eosin-Y	DTBP	DMSO/H_2_O	12	21
8	Eosin-Y	H_2_O_2_	DMSO/H_2_O	12	Trace
9	Eosin-Y	Na_2_S_2_O_8_	DMSO/H_2_O	12	62
10	Eosin-Y	K_2_S_2_O_8_	DMSO/H_2_O	10	64
11	Eosin-Y	K_2_S_2_O_8_	DMSO/H_2_O	15	76
12	Eosin-Y	K_2_S_2_O_8_	DMSO	12	56
13	Eosin-Y	K_2_S_2_O_8_	DCE	12	49
14	Eosin-Y	K_2_S_2_O_8_	CH_3_CN	12	38
15	Eosin-Y	K_2_S_2_O_8_	DMF	12	25
16	Eosin-Y	K_2_S_2_O_8_	CH_2_Cl_2_	12	23
17	Eosin-Y	K_2_S_2_O_8_	MeOH	12	29
18	Eosin-Y	K_2_S_2_O_8_	EtOH	12	35
19^c^	—	K_2_S_2_O_8_	DMSO/H_2_O	10	56

a Reaction conditions: 1 (0.6 mmol), 2 (0.2 mmol), oxidant (0.6 mmol), photocatalyst (5 mol%), solvent (2 mL).

b Isolated yield.

c Reaction at 50 °C in the absence of photocatalyst and light.

**Fig. 3:**
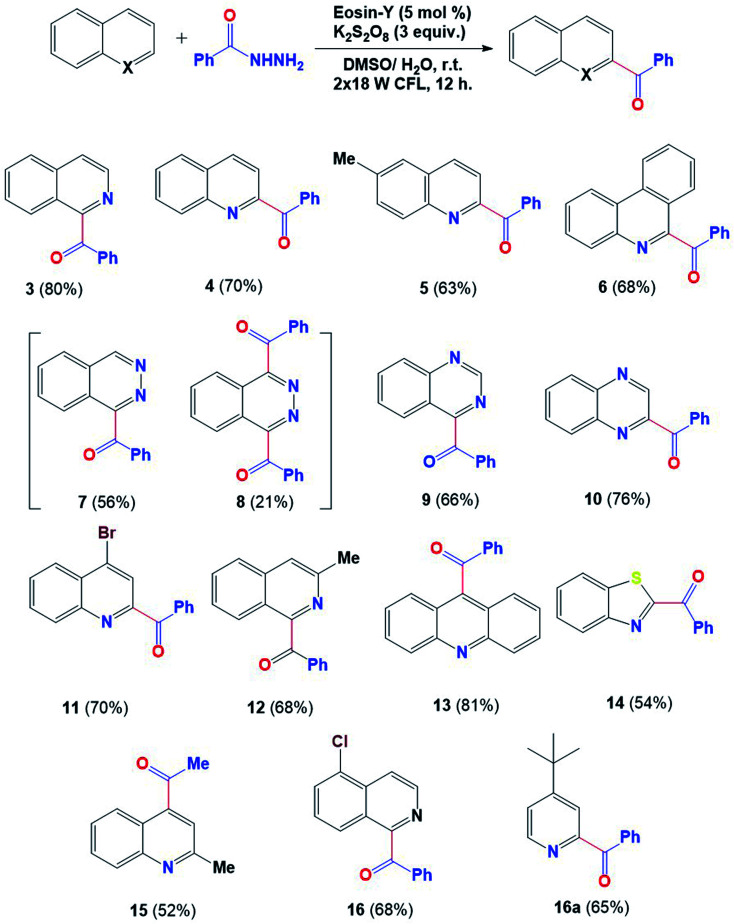
Scope of heteroarenes. ^a^Reaction conditions: benzoyl hydrazine (0.6 mmol), heteroarene (0.2 mmol), oxidant (0.6 mmol), solvent DMSO/H_2_O (4 : 1) 2 mL under 36 W (2 CFL of 18 W each).

Having the optimized reaction conditions in hand, as indicated in Table 1 entry 3, the scope of this dehydrazinative C–H acylation was then explored with various N-heterocycles. A variety of N-heterocycles, including isoquinoline, quinoline, phenanthridine, dihydroacridine, quinoxaline and phthalazine, underwent dehydrazinative C–H acylation to produce acylated heterocycles with moderate to high yields, as shown in Fig. 3. High regioselectivity was observed in the case of quinazoline (9), quinoxaline (10) and acridine (13) while in the case of phthalazine, a monoacylated compound (7) was detected with 56% yield along with a diacylated product (8) with 21% yield. The benzothiazole moiety may also be expanded using this protocol, furnishing its acylated product (14) with 54% yield. 2-Methylquinoline showed effective coupling with aliphatic hydrazide to give the acylated product (15) with 52% yield under optimized reaction conditions. No acylated product is formed in the case of pyridine and pyrazine under this protocol; however, *tert*-butyl pyridine furnished the acylated product with 65% yield (16a).

**Fig. 4:**
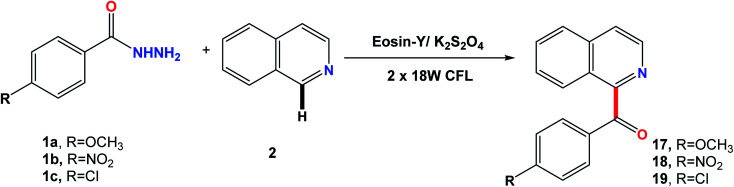
Scope of acyl hydrazides.

Extending the scope of the reaction with respect to the phenyl ring of 1 substituted with electron donating and withdrawing groups (1a–1c) showed that it worked well and could tolerate various substituents on the ring to afford 17–19. The reaction proceeded with good selectivity and yields, using the optimized conditions mentioned above (Fig. 4).

**Fig. 5:**
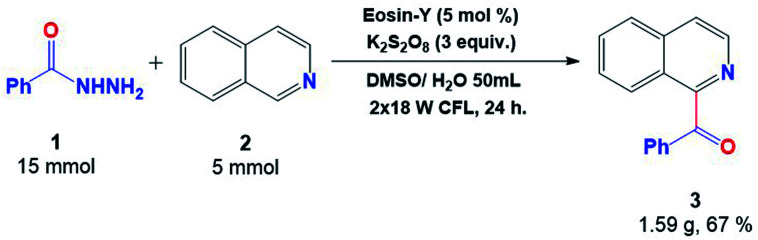
Gram-scale synthesis of 3.

The reaction was performed on a large scale in order to elaborate the utility and applicability of this protocol. The gram-scale reaction proceeded effectively well towards the synthesis of the acylated product (3) with 67% yield, clearly indicating its ability to be useful in the protocol (Fig. 5).

**Fig. 6:**
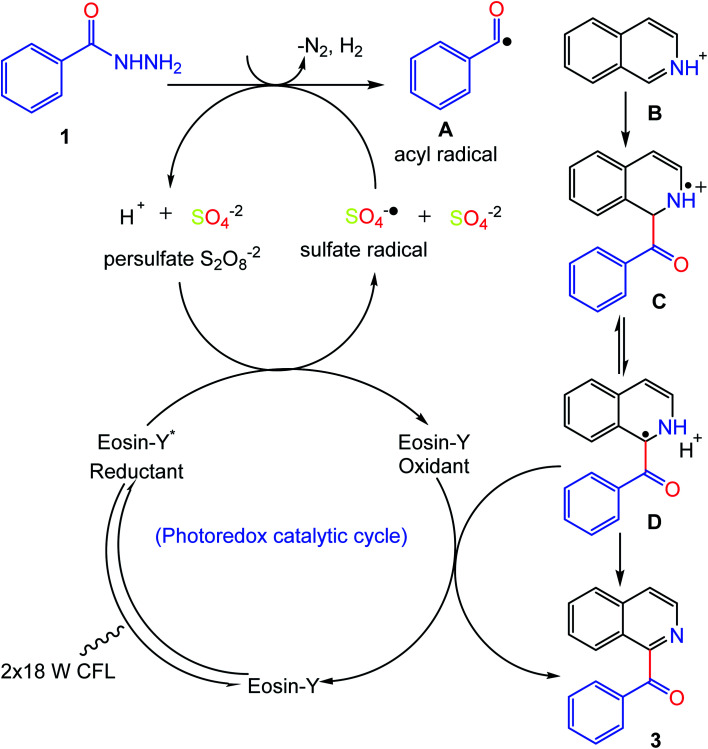
Proposed reaction pathway.

Based on and in comparison with previous literature reports,^22,29,30,32^ a proposed reaction mechanism for this photocatalytic transformation was developed and is shown in Fig. 6. Possibly, the reaction starts with the excitation of the photocatalyst which undergoes single electron transfer (SET) in the presence of the persulfate salt, generating the sulfate radical anion (SO_4_^−^˙) and (eosin-Y oxidant). Meanwhile, benzoyl hydrazine (1) undergoes a removal of nitrogen and hydrogen followed by hydrogen atom transfer (HAT) with the sulfate radical anion (SO_4_^−^˙) to produce the acyl radical (A). Consequently, the acyl radical couples with the protonated heteroarene B to give the intermediates C and D. The final product 3 is produced by SET between intermediate D and the (eosin-Y oxidant), thus completing the photo-redox catalytic cycle by regeneration of the photocatalyst.

**Fig. 7:**
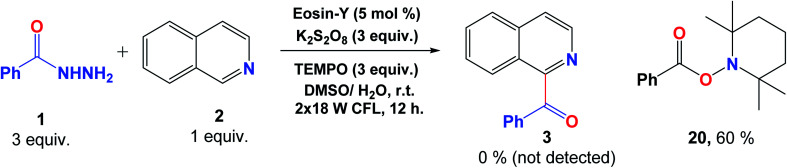
Control experiment confirming radical formation for this transformation.

To confirm the proposed mechanism of this photocatalytic transformation, a reaction between benzoyl hydrazine (1) and isoquinoline (2) was conducted in the presence of the radical scavenger TEMPO. The desired acylated product 3 was not observed while the radical capture product 2,2,6,6-tetramethylpiperidino benzoate (20) was detected with 60% yield (Fig. 7). This observation strongly supports the proposal of the formation of radical intermediates in this reaction.

## Conclusion

In conclusion, we have developed a visible light-induced acylation of N-heterocycles using benzoyl hydrazides as an efficient acyl source under mild reaction conditions. Photo-redox catalyzed oxidative cleavage of hydrazides leads to *in situ* formation of acyl radicals, which subsequently couple with various N-heterocycles to produce acylated products. This synthetic strategy performs the classic Minisci reaction in an eco-friendly and greener way with functional group tolerance and regioselectivity. Control experiments confirm the radical pathway for this transformation.

## Conflicts of interest

The authors declare no conflict of interest.

## Supplementary Material

RA-011-D1RA07063K-s001
