# Supplementary material for: Photo-redox catalyzed dehydrazinative acylation of N-heterocycles via Minisci reaction
Source: RSC Adv. 2021 Dec 1;11(61):38683–9. doi: 10.1039/d1ra07063k (PMC9044185; doi:10.1039/d1ra07063k)
Supplement: RA-011-D1RA07063K-s001 [file RA-011-D1RA07063K-s001.pdf]

Electronic Supplementary Material (ESI) for RSC Advances.
This journal is © The Royal Society of Chemistry 2021

Supporting information

Photo-redox Catalyzed Dehydrazinative Acylation of *N*-heterocycles via Minisci Reaction.

Saira Hafeez[a] and Aamer Saeed[b*]

[a]Department of Chemistry, University of Science and Technology of China, Hefei, Anhui 230026, China

[b]Department of Chemistry, Quaid-I-Azam University, 45320, Islamabad, Pakistan

*Corresponding Author:

Prof. Dr. Aamer Saeed; aamersaeed@yahoo.com, asaeed@qau.edu.pk

Table of Contents

General Information………………………………………………………... S-3

Experimental Procedure………………………………………………………. S-3

Experimental Procedure for Scaling-up Reaction.……………………………. S-3

Characterization Data of the Reaction Products.……………………………… S-4

^{1}H NMR Spectra of the Synthetic Products.……………………………...... S-11

General information

All chemicals were purchased from commercial suppliers like Meryer, Acros, Alfa Aesar, and TCI and used as delivered without any further purification.

^{1}H NMR spectra were recorded on a Bruker AC-400 FT spectrometer (400 MHz) using TMS as an internal standard. NMR multiplicities are abbreviated as follows: s = singlet, d = doublet, t = triplet, m = multiplet and q = quartet. Chemical shifts (δ) are recorded on a LC-TOF spectrometer (Micromass) in ppm while coupling constants *(J)* are expressed in Hz.

General experimental procedure for photo-redox catalyzed dehydrazinative acylation of *N*-heterocycles

To an oven dried flask containing a magnetic stirrer, heteroarene (0.2 mmole, 1 equiv.), benzoyl hydrazine (0. 6 mmole, 3 equiv.), potassium persulfate (0.6 mmole. 3 equiv.), Eosin-Y (5 mol %, 0.01 equiv.) and DMSO/H_2O (4:1) 2mL were added. The resulting mixture was kept stirring for 12 h under an irradiation of 2×18 W CFL bulb (at a distance of 5 cm from the flask) in opposite direction. After indicated time, EtOAc was added and the mixture is washed with $NaHCO_3$ three times and aqueous layer was separated. The combined organic layer was washed with brine, drying over sodium sulfate, solvent removed under reduced pressure and then mixture is subjected to silica gel chromatography to get the pure desired product.

Experimental procedure for Scaling-up reaction

To an oven dried 100 mL round bottom flask containing a magnetic stirrer bar, heteroarene (5 mmole, 1 equiv.), benzoyl hydrazine (15 mmole, 3 equiv.), potassium persulfate (15 mmole, 3 equiv.), Eosin-Y (0.25 mmole, 5 mol %) and DMSO/H_2O (4:1) 50 mL were added. The resulting mixture was kept stirring for 24 h under an irradiation of 2×18 W CFL bulb (at a distance of 5 cm

from the flask) in opposite direction. After indicated time, EtOAc was added and the mixture is washed with $NaHCO_3$ three times and aqueous layer was separated. The combined organic layer was washed with brine, drying over sodium sulfate, solvent removed under reduced pressure and then mixture is subjected to silica gel chromatography to get the pure desired product.

Characterization data of the Synthesized Products

Isoquinolin-1-yl(phenyl)methanone **(3)**

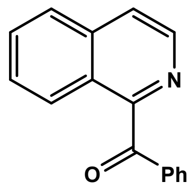

3

Prepared as described in the general experimental procedure. Brown solid; Yield 80%; m.p. 74-77 °C (lit.[36] 74-75 °C); IR (KBr, cm^{-1}) 2923, 1670; ^1H NMR (400 MHz, $CDCl_3$): δ 8.55 (d, *J = 4 Hz*, 1H), 8.15 (*d, J = 8 Hz*, 1H), 7.90-7.85 (t, *J = 10 Hz*, 3H), 7.76 (d, *J = 4 Hz*, 1H), 7.71-7.66 (t, *J = 8 Hz*, 1H), 7.59-7.52 (q, *J = 6.7 Hz*, 2H), 7.44-7.38 (t, *J = 8 Hz*, 2H); ^{13}C NMR (100 MHz, $CDCl_3$)[22,36]. δ 194.7, 156.4, 141.1, 136.7, 136.6, 133.7, 130.7, 130.4, 128.5, 128.3, 127.1, 126.4, 126.2, 122.6; HRESI-MS (m/z) Calculated for $C_{16}H_{11}NONa$ (M+Na) 256.0738 found (M+Na) 256.0742. ^1H NMR data is consistent with the reported literature values[22,30,32].

Phenyl(quinolin-2-yl)methanone **(4)**[40]

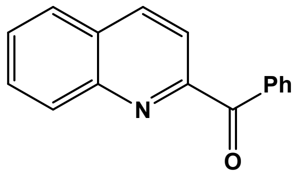

4

Prepared as described in the general experimental procedure as yellow viscous oil. ^{1}H NMR (400 MHz, $CDCl_3$) δ 8.35 (d, *J = 8.5 Hz*, 1H), 8.22 (dd, *J = 14.4, 8.1 Hz*, 3H), 8.11 (d, *J = 8.5 Hz*, 1H), 7.91 (d, *J = 8.1 Hz*, 1H), 7.79 (t, *J = 7.6 Hz*, 1H), 7.72 – 7.57 (m, 2H), 7.52 (t, *J = 7.6 Hz,* 2H). ^{13}C NMR (150 MHz, $CDCl_3$) δ 193.97, 154.83, 146.85, 137.25, 136.27, 133.25, 131.60, 130.70, 130.25, 129.05, 128.58, 128.30, 127.77,120.97; IR[22] (KBr) 3054, 2925, 2853, 1661, 1450, 1317, 1168, 1019 cm^{-1}; HRMS (ESI) calculated for $C_{16}H_{11}NO$ (MH^+) 234.0913, found 234.0917.

(6-Methylquinolin-2-yl)(phenyl)methanone **(5)**[40]

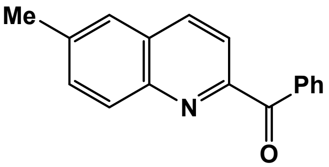

5

Prepared as described in the general procedure as yellow viscous oil. ^{1}H NMR (400 MHz, $CDCl_3$) δ 8.24 (dd, *J = 7.9, 2.9 Hz,* 3H), 8.09 (d, *J = 8.4 Hz*, 2H), 7.66 (s, 1H), 7.65 – 7.58 (m, 2H), 7.51 (t, *J = 7.6 Hz,* 2H), 2.59 (s, 3H). ^{13}C NMR (150 MHz, $CDCl_3$) δ 194.01, 153.97, 145.46, 138.88, 136.45, 136.41, 133.09, 132.58, 131.59, 130.35, 129.13, 128.25, 126.61, 121.03, 21.95. IR[22] (KBr) 3056, 2953, 2922, 1660, 1594, 1445, 1322, 1286, 1159, 1070 cm^{-1}; HRMS (ESI) calculated for $C_{17}H_{13}NO$ (MH^+) 248.1070, found 248.1078.

Phenanthridin-6-yl(phenyl)methanone **(6)**[40]

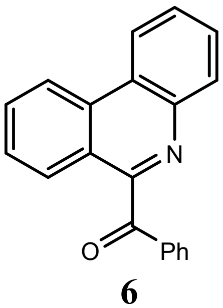

6

Prepared as described in the general experimental procedure. Colorless liquid; Yield 68%; IR[37] (Neat, cm^{-1}) 2927, 1673; ^{1}H NMR (400 MHz, $CDCl_3$):δ 8.72 (d, *J = 8.47 Hz*, 1 H), 8.65 (d, *J = 8.08 Hz*, 1 H), 8.25 (d, *J = 7.40 Hz*, 1 H), 8.18 (d, *J = 8.18 Hz*, 1 H), 8.01 (d, *J = 8.05 Hz*, 2 H), 7.87-7.84 (m, 1 H), 7.67-7.63 (m, 2 H), 7.62-7.56 (m, 2 H), 7.50-7.45 (m, 2 H); ^{13}C NMR (100 MHz, $CDCl_3$):δ 195, 155.4, 141.6, 136.5, 134, 133.6, 131.1, 126.2, 131.3, 130.7, 129.5, 128.2, 128.5, 128, 127.6, 127.1, 124, 123.5, 122.3, 122; HRESI-MS (m/z) Calculated for $C_{20}H_{13}NONa$ (M+Na) 306.0895 found (M + Na) 306.0895.

Phthalazine-1-yl-(phenyl)methanone **(7)**[30]

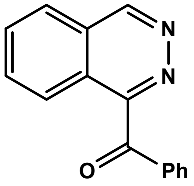

7

Prepared as described in the general experimental procedure. White solid; 56% yield. ^{1}H NMR (400 MHz, $CDCl_3$) δ (ppm): 9.55 (s, 1H), 8.23 (dd, *J = 7.8, 1.2 Hz,* 1H), 8.1 (d, *J = 7.8 Hz*, 1H), 8.03 (d, *J = 7.8 Hz*, 2H), 7.90 – 7.83 (m, 2H), 7.58 (t, *J = 7.4 Hz,* 1H), 7.42 (t, *J = 7.4 Hz*, 2H). ^{13}C NMR (100 MHz, $CDCl_3$) δ (ppm): 192.7, 156.2, 152.1, 136.2, 134.4, 133.6, 133.3, 131.7, 128.9, 127.4, 127.1, 125.2, 125. IR[30] (cm^{-1}): 3061, 2924, 1668, 1596, 1449, 1224, 931, 759, 714, 645. HRMS (ESI): m/z calcd for: $C_{15}H_{11}N_2O$ [M+H]+: 235.0866, Found: 235.0869.

Phthalazine-1,4-diylbis(phenyl)methanone **(8)**[30]

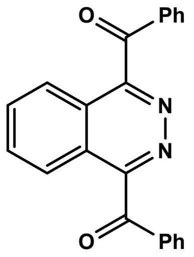

8

Prepared as described in the general experimental procedure. White solid; 21% yield. ^{1}H NMR (400 MHz, $CDCl_3$) δ (ppm): 8.30 (dd, J = *6.3, 3.2 Hz*, 2H), 8.1 – 8.06 (m, 4H), 7.91 (dd, *J = 6.3, 3.2 Hz, 2H*), 7.64 – 7.60 (m, 1H), 7.55 (t, *J = 7.8 Hz*, 4H). ^{13}C NMR (100 MHz, $CDCl_3$) δ (ppm): 192.8, 157, 136.1, 134.8, 133.5, 131.3, 129, 125.8, 125.6. IR[30] (cm^{-1}): 2923, 1666, 1596, 1450, 1242, 1229, 918, 768, 710, 681. HRMS (ESI): m/z calcd for: $C_{22}H_{15}N_2O_2$ [M+H]+: 339.1128, Found: 339.1131.

4-benzoylquinazoline (**9**)

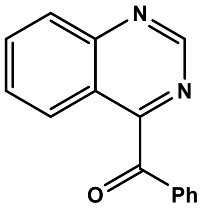

9

Prepared as described in the general experimental procedure. White solid; 56% yield. ^{1}H NMR (400 MHz, $CDCl_3$) δ (ppm): 9.40 (s, 1H), 8.15 (d, *J = 8.6 Hz*, 1H), 8.06 (d, *J = 8.6 Hz*, 1H), 7.98 – 7.92 (m, 3H), 7.66 – 7.60 (m, 2H), 7.50 (t, *J = 7.8 Hz*, 2H). ^{13}C NMR (100 MHz, $CDCl_3$) δ (ppm): 193.18, 164.32, 154.02, 151.45, 135.40, 134.87, 134.7,3 130.85, 129.27, 129, 128.95, 126.08, 122.30. IR νmax (cm^{-1}): 3061, 2926, 1672, 1550, 1492, 1450, 1278, 1226, 927, 766, 716, 637. Data are consistent with reported literature values [30].

Phenyl(quinoxalin-2-yl) methanone **(10)**[30]

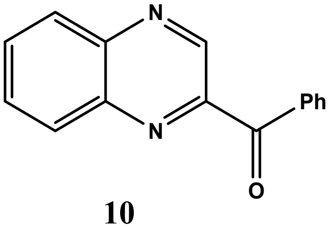

10

Prepared as described in the general experimental procedure. Colorless liquid; Yield 76%; IR[36] (KBr, cm^{-1}) 2928, 1726; ^{1}H NMR (400 MHz, $CDCl_3$): δ 9.52 (s, 1 H), 8.23-8.20 (m, 3 H) 8.21-8.18 (m, 1 H), 7.90-7.85 (m, 2 H), 7.72-7.66 (m, 1 H), 7.52-7.48 (m, 2 H); ^{13}C NMR (100 MHz, $CDCl_3$): δ 192, 148.3, 145.6, 143.1, 140.7, 135.2, 133.9, 132.1, 131.6, 130.6, 130.2, 129, 128.4; HRESI-MS (m/z) Calculated for $C_{15}H_{10}N_2ONa$ (M+Na) 257.0691 found (M+Na) 257.0693.

(4-Bromoisoquinolin-1-yl) (phenyl)methanone **(11)**[22,23]

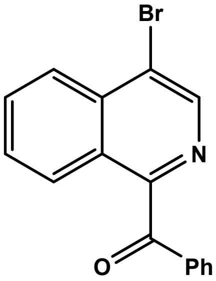

11

Prepared as described in the general experimental procedure. White solid; Yield 70%; m.p: 127 °C (lit.[11] 129-130 °C) ; IR[23] (KBr, cm^{-1}) 2962, 1666; ^{1}H NMR (400 MHz, $CDCl_3$) δ 8.80 (s, 1H), 8.28 (d, *J = 8.5 Hz*, 1H), 8.23 (d, *J = 8.5 Hz*, 1H), 8.00 – 7.91 (m, 2H), 7.86 (ddd*, J = 8.4, 6.9, 1.1 Hz,* 1H), 7.68 (ddd, *J = 8.2, 7.0, 1.1 Hz*, 1H), 7.65 – 7.57 (m, 1H), 7.48 (dd, *J = 10.6, 4.8 Hz,* 2H). ^{13}C NMR (150 MHz, $CDCl_3$) δ 194.07, 155.78, 143.03, 143.01, 136.46, 135.61, 134.02, 132.13,

130.85, 129.34, 128.65, 127.66, 126.74, 126.58, 121.89. HRMS (m/z) Calculated for $C_{16}H_{10}BrNONa$ (M+Na) 333.984 found (M+Na) 333.981.

(3-Methylisoquinolin-1-yl)(phenyl)methanone **(12)**[23]

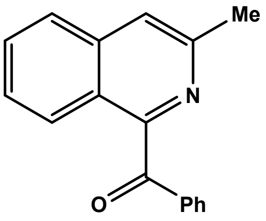

12

Prepared as described in the general experimental procedure. Yield 76%. brown solid; yield 64; m.p. 94−96 °C. ^{1}H NMR (400 MHz, $CDCl_3$) δ 8.06 (d, *J = 8.5 Hz,* 1H), 7.96 (d, *J = 7.6 Hz*, 2H), 7.80 (d, *J = 8.3 Hz*, 1H), 7.65 (t, *J = 7.5 Hz*, 1H), 7.63 – 7.55 (m, 2H), 7.53 – 7.41 (m, 3H), 2.72 (s, 3H). ^{13}C NMR (150 MHz, $CDCl_3$) δ 195.01, 156.35, 150.36, 137.40, 136.52, 133.82, 130.94, 130.65, 128.53, 127.26, 126.59, 126, 124.48, 120.55, 24.22. IR[22] (KBr) 3059, 2924, 2855, 1670, 1589, 1447, 1239, 1027 cm^{-1}; HRMS (ESI) calcd. for $C_{17}H_{13}NO$ (MH^{+}) 248.1070, found 248.1073.

Acridin-9-yl(phenyl)methanone (**13**)[30]

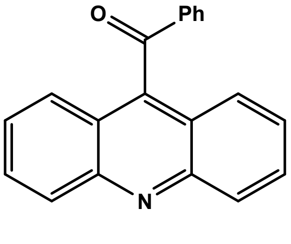

13

Prepared as described in the general experimental procedure. Pale yellow solid, yield 81%. ^{1}H NMR (400 MHz, $CDCl_3$) *δ* (ppm): 8.30 (d, *J = 8.8 Hz*, 2H), 7.81 – 7.76 (m, 4H), 7.71 (d, *J = 8.8 Hz*, 2H), 7.64 – 7.59 (m, 1H), 7.48 – 7.41 (m, 4H). ^{13}C NMR (100 MHz, $CDCl_3$) *δ* (ppm): 198,

148.6, 143.7, 137.3, 134.6, 130.5, 130.2, 130, 129.4, 127, 125.7, 123.2. IR[30] (cm^{-1}): 3058, 2925, 1660, 1448, 1233, 899, 747, 705, 622. Data are consistent with reported literature values [3].

2-benzoyl-1,3-benzothiazole (**14**)[30]

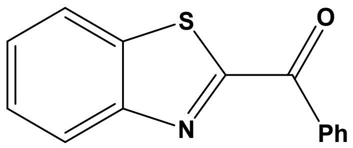

14

Prepared as described in the general experimental procedure. Pale yellow solid; 54% yield. ^1H NMR (400 MHz, $CDCl_3$) δ (ppm): 8.54 – 8.50 (m, 2H), 8.35 – 8.32 (m, 1H), 8.08 – 8.006 (m, 1H), 7.60 – 7.56 (m, 1H), 7.56 – 7.50 (m, 4H). ^{13}C NMR (100 MHz, $CDCl_3$) δ (ppm): 185.6, 167.4, 154.1, 137.3, 135.1, 134, 131.6, 128.8, 127.5, 127.1, 126, 122.3. IR[30] (cm^{-1}): 3062, 1644, 1481, 1287, 1272, 1120, 888, 759, 706, 671. Data are consistent with reported literature values [12].

1-(2-methylquinolin-4-yl)ethane-1-one (**15**)[30]

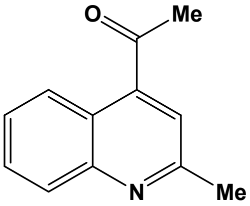

15

Prepared as described in the general experimental procedure. White solid; 52% yield. ^1H NMR (400 MHz, $CDCl_3$) δ (ppm): 8.32 (dd, J = *8.6*, 1.0 Hz, 1H), 8.05 (dd, J = *8.6,* 1.0 Hz, 1H), 7.70 – 7.66 (m, 1H), 7.55 – 7.50 (m, 1H), 7.40 (s, 1H), 2.75 (s, 3H), 2.70 (s, 3H). ^{13}C NMR (100 MHz, $CDCl_3$) δ (ppm): 201.60, 158.65, 148.95, 143.32, 130.10, 129.28, 127.58, 125.39, 122.10, 120.95,

30.35, 25.52. IR vmax (cm^{-1}): 2923, 2853, 1689, 1593, 1356, 1326, 1237, 1182, 1146, 764, 608. Data are consistent with reported literature values [30].

(5-Chloroisoquinolin-1-yl)(phenyl)methanone **(16)**[22,23]

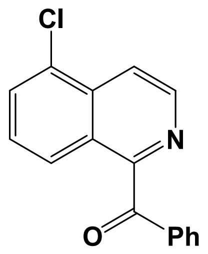

16

Prepared as described in the general experimental procedure. Brown solid; Yield 68%; m.p 104 °C; brownish solid.^{1}H NMR (400 MHz, $CDCl_3$) δ 8.71 (d, *J = 5.9 Hz*, 1H), 8.22 (d, *J = 5.9 Hz*, 1H), 8.14 (d, *J = 8.5 Hz,* 1H), 8.01 – 7.89 (m, 2H), 7.82 (dd, *J = 7.5, 0.7 Hz,* 1H), 7.62 (t, *J = 7.4 Hz*, 1H), 7.57 – 7.43 (m, 3H). ^{13}C NMR (150 MHz, $CDCl_3$) δ 194.45, 156.86, 142.42, 136.44, 134.65, 134, 131.65, 130.86, 130.80, 128.65, 128.25, 127.39, 125.35, 119. IR[23] (KBr) 3066, 2922, 2844, 1661, 1526, 1353, 1276, 1161, 1069 cm^{-1}; HRMS (ESI) calcd for $C_{16}H_{10}N_2O_3$ (MH^+) 279.0764, found 279.0768.

(4-(tert-butyl)pyridin-2-yl)(phenyl)methanone (**16a**)[23]

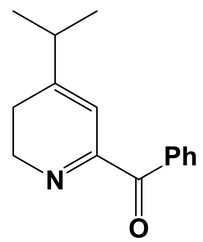

16a

Prepared as described in the general experimental procedure as yellow viscous oil. ^{1}H NMR (400 MHz, $CDCl_3$) δ 8.62 (d, *J = 5.2 Hz*, 1H), 8.13 – 7.99 (m, 3H), 7.58 (t, *J = 7.4 Hz*, 1H), 7.48 (dd, *J*

= *8.3, 5.0 Hz*, 3H), 1.38 (s, 9H). ^{13}C NMR (150 MHz, $CDCl_3$) δ 194.65, 161.55, 155.18, 148.61, 136.67, 132.97, 131.15, 128.25, 123.45, 121.80, 35.20, 30.65. Analytical data are consistent with those reported previously.

Isoquinolinyl(4-methoxyphenyl)methanone (**17**)[41]

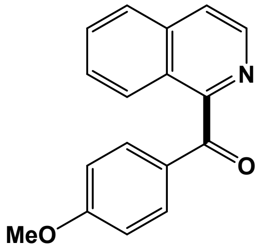

17

Prepared as described in the general experimental procedure. Pale yellow oily liquid; Yield 56%; IR (Neat, cm^{-1}) 3055, 2931, 2840, 1663, 1597, 1251, 1152; ^{1}H NMR (400 MHz, $CDCl_3$) δ (ppm) 8.59 (d, *J = 5.6 Hz,* 1H), 8.17 (d*, J = 8.4 Hz*, 1H), 7.96−7.89 (m, 3H), 7.78 (d, *J = 5.6 Hz*, 1H), 7.75−7.70 (m, 1H), 7.61−7.57 (m, 1H), 6.94 (d, *J = 8.8 Hz*, 2H), 3.86 (s, 3H); ^{13}C NMR (100 MHz, $CDCl_3$) δ (ppm) 193.4, 164.1, 157.1, 141.2, 136.6, 133.1, 130.6, 129.5, 128.1, 127.0, 126.3, 126.3, 122.2, 113.8, 56.1.

Isoquinolinyl(4-nitrophenyl)methanone (**18)**

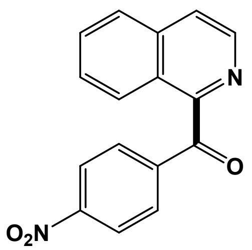

18

Prepared as described in the general experimental procedure. Pale yellow solid; 47% yield. ^{1}H NMR (300 MHz, DMSO-d6) δ: 8.99 (s, 1H), 8.60 (s, 1H), 8.46 – 8.14 (m, 4H), 7.82 (d, *J = 7.9*

Hz, 2H), 7.64 (d, *J* = *6.6 Hz,* 2H). ^{13}C NMR (75 MHz, DMSO-d6) δ: 192.73, 150.33, 149.22, 141.39, 141.19, 136.16, 130.73, 130.73, 130.33, 128.63, 127.56, 126.38, 126.31, 125.19, 123.25, 123.25.

4-Chlorophenyl(isoquinolinyl)methanone **(19)**

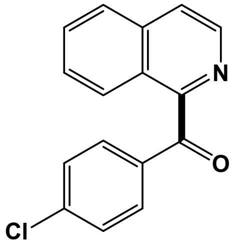

19

RSC Advances Page 30 of 40 S-11 Prepared as described in the general experimental procedure. Pale yellow solid; 54% yield. ^{1}H NMR (300 MHz, DMSO-d6) δ: 8.95 (s, 1H), 8.60 (s, 1H), 8.07 – 7.93 (m, 2H), 7.81 (d, *J* = *8.1 Hz*, 2H), 7.64 (d, *J* = *4.7 Hz*, 2H), 7.48 – 7.34 (m, 2H). ^{13}C NMR (75 MHz, DMSO-d6) δ: 192.73, 149.22, 141.39, 140.25, 136.16, 135.30, 131.44, 131.44, 130.33, 128.76, 128.76, 128.63, 127.56, 126.38, 126.31, 125.19.

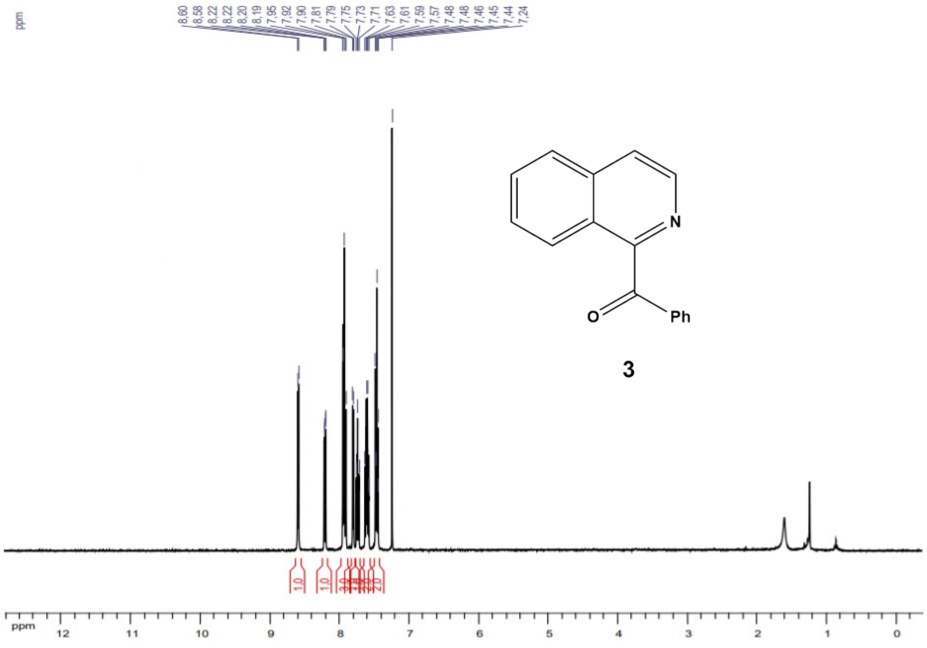

^{1}H NMR of Isoquinolin-1-yl(phenyl)methanone **(3)**

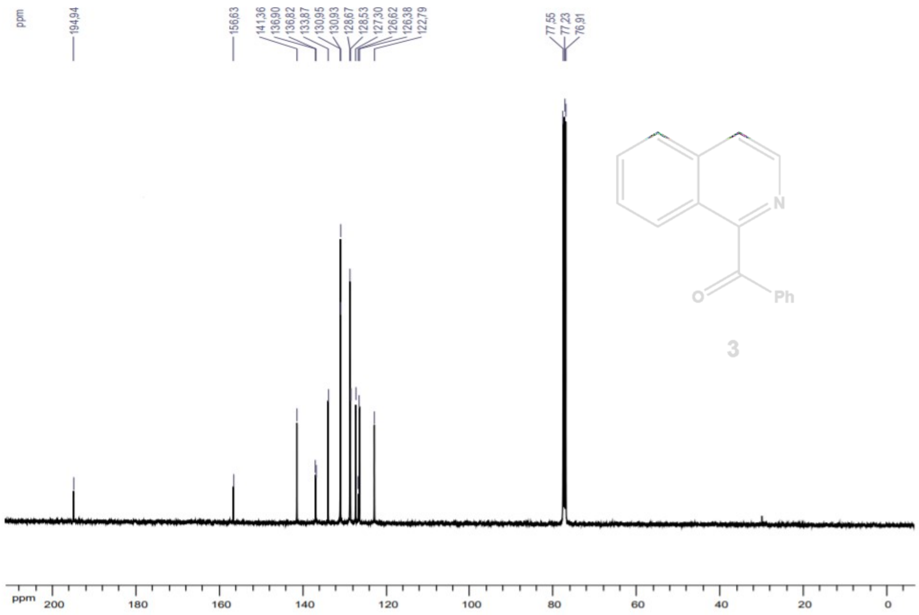

^{13}C NMR of Isoquinolin-1-yl(phenyl)methanone **(3)**

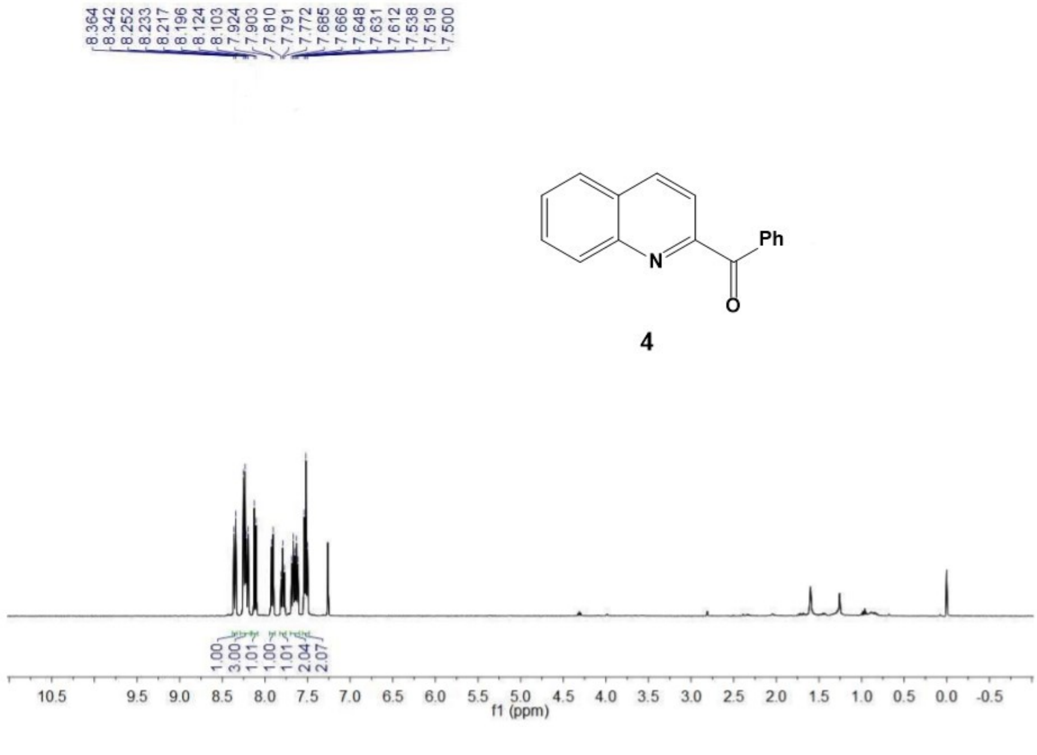

^{1}H NMR of Phenyl(quinolin-2-yl)methanone **(4)**

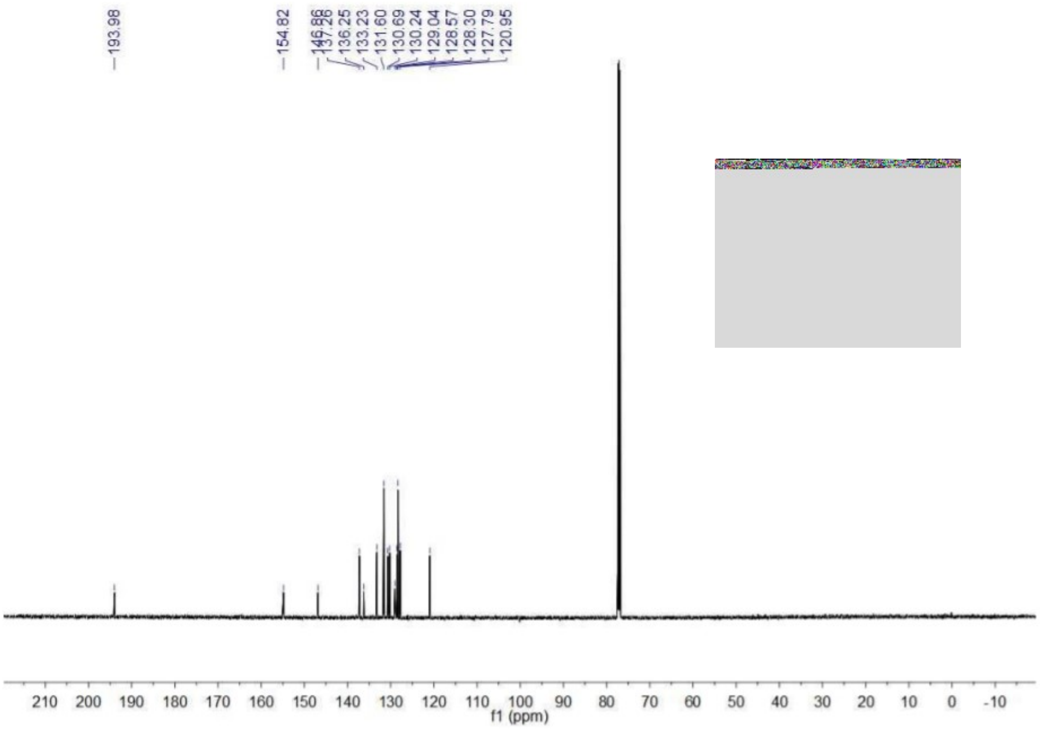

^{13}C NMR of Phenyl(quinolin-2-yl)methanone **(4)**

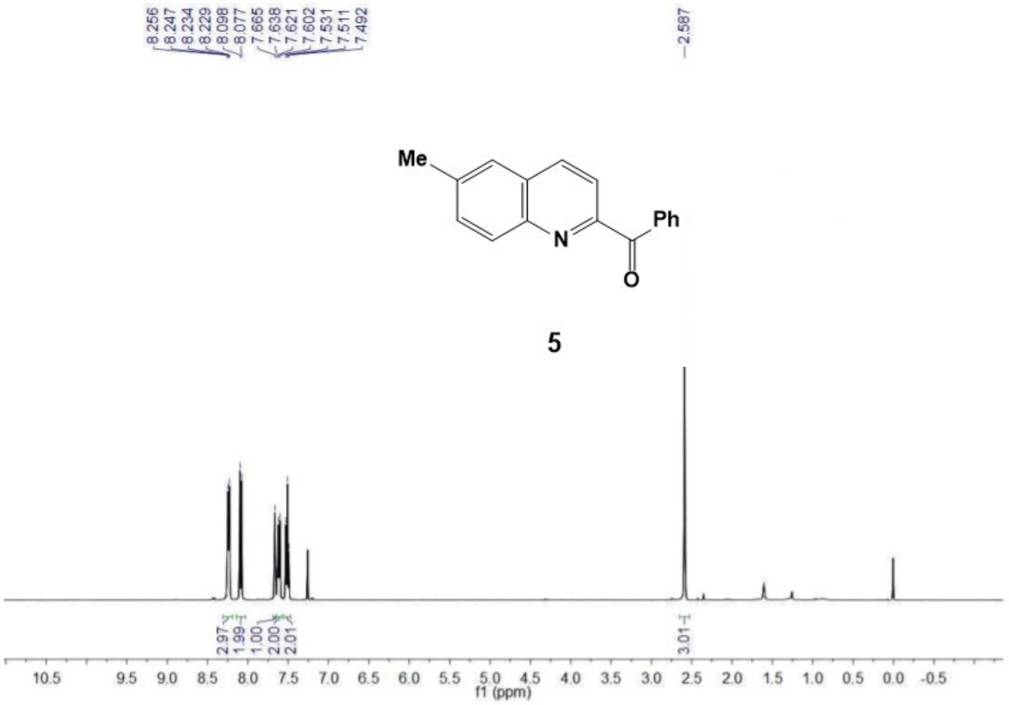

^{1}H NMR of (6-Methylquinolin-2-yl)(phenyl)methanone **(5)**

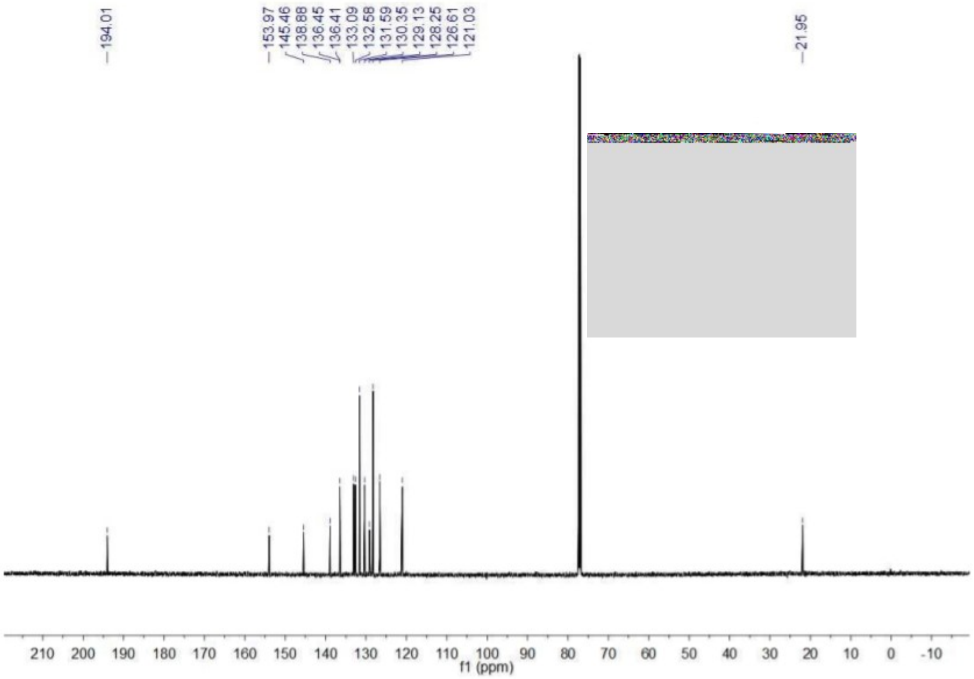

^{13}C NMR of (8-Methylquinolin-2-yl)(phenyl)methanone **(5)**

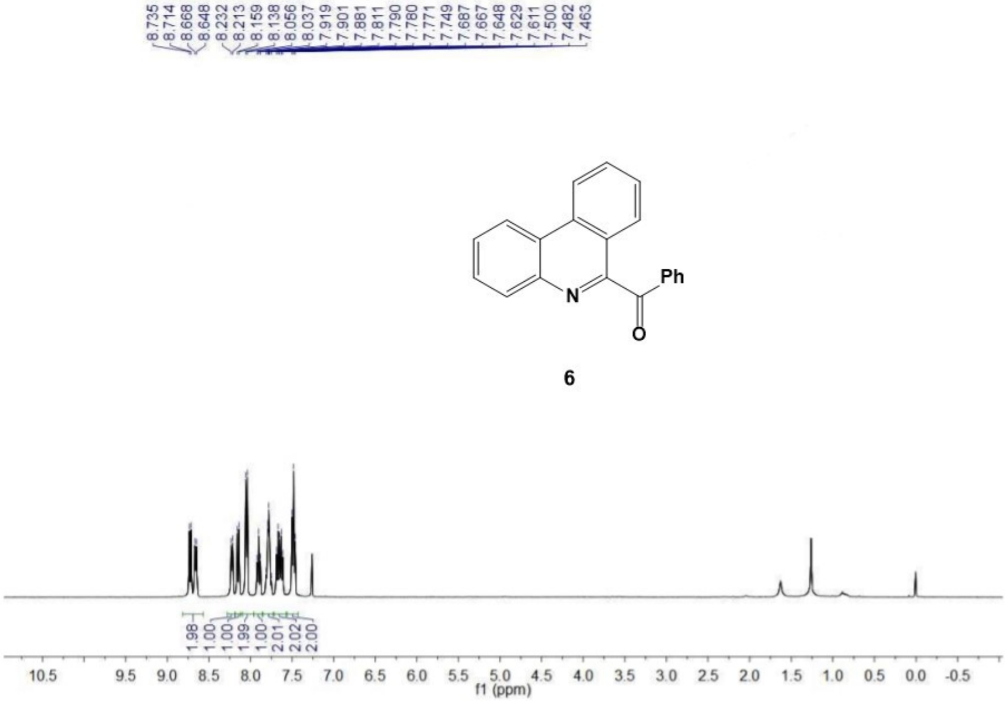

^{1}H NMR of Phenanthridin-6-yl(phenyl)methanone **(6)**

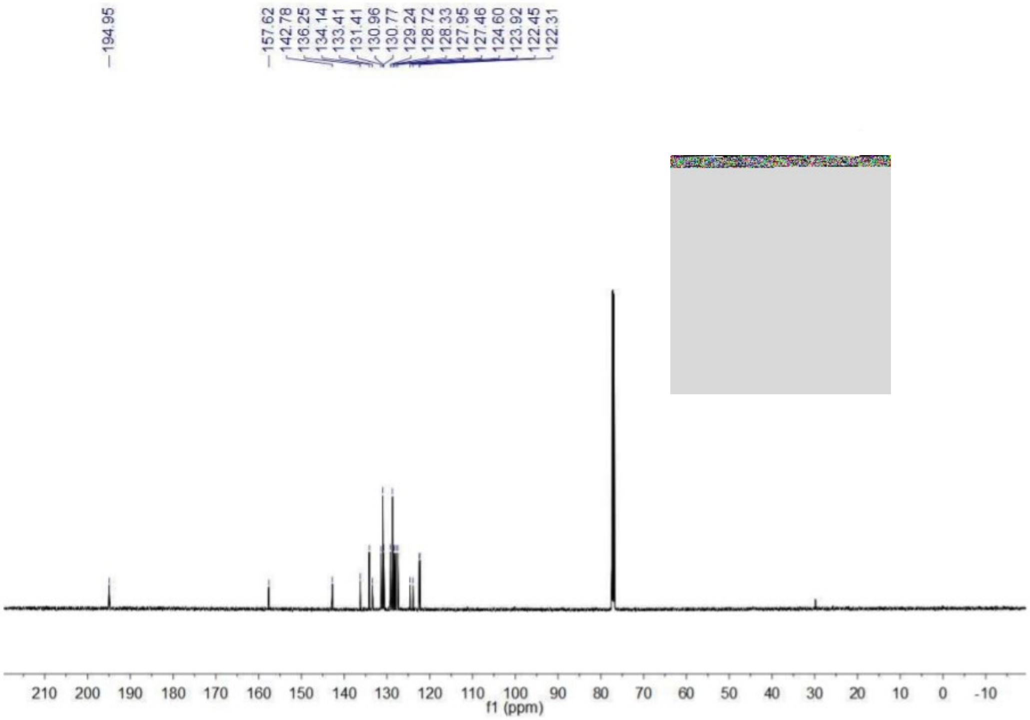

^{13}C NMR of Phenanthridin-6-yl(phenyl)methanone **(6)**

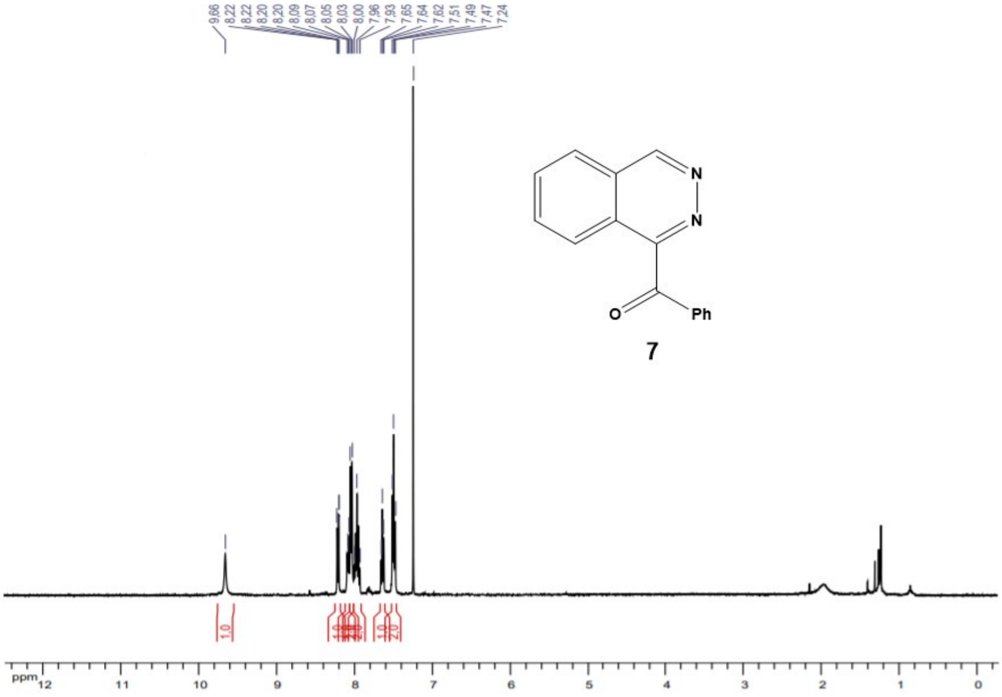

^{1}H NMR of Phthalazine-1-yl-(phenyl)methanone **(7)**

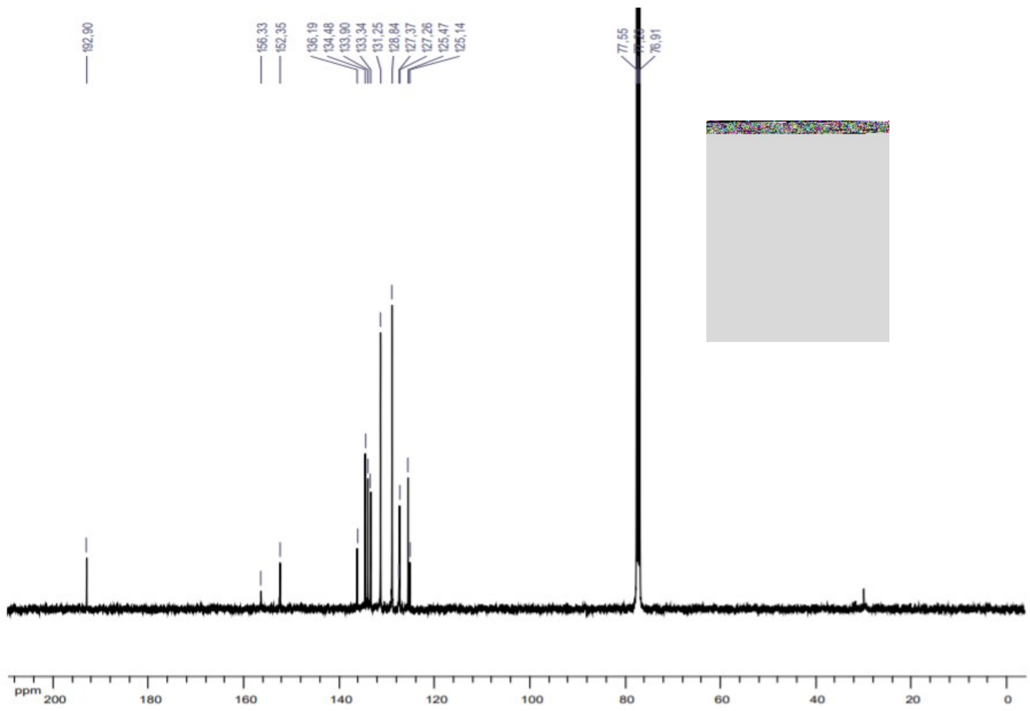

^{13}C NMR of Phthalazine-1-yl-(phenyl)methanone **(7)**

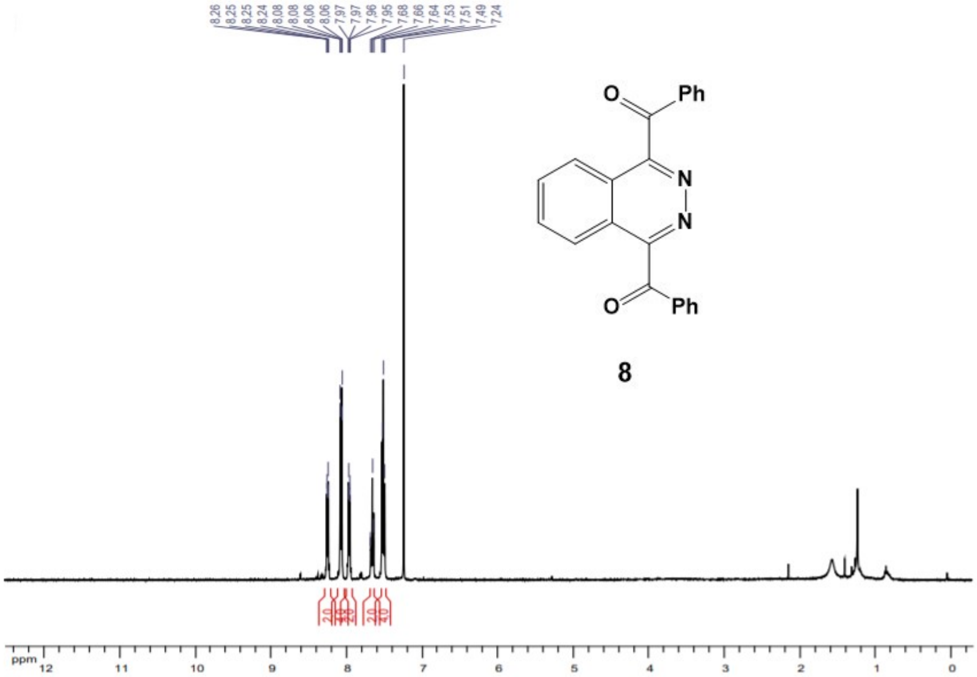

^{1}H NMR of Phthalazine-1,4-diylbis(phenyl)methanone **(8)**

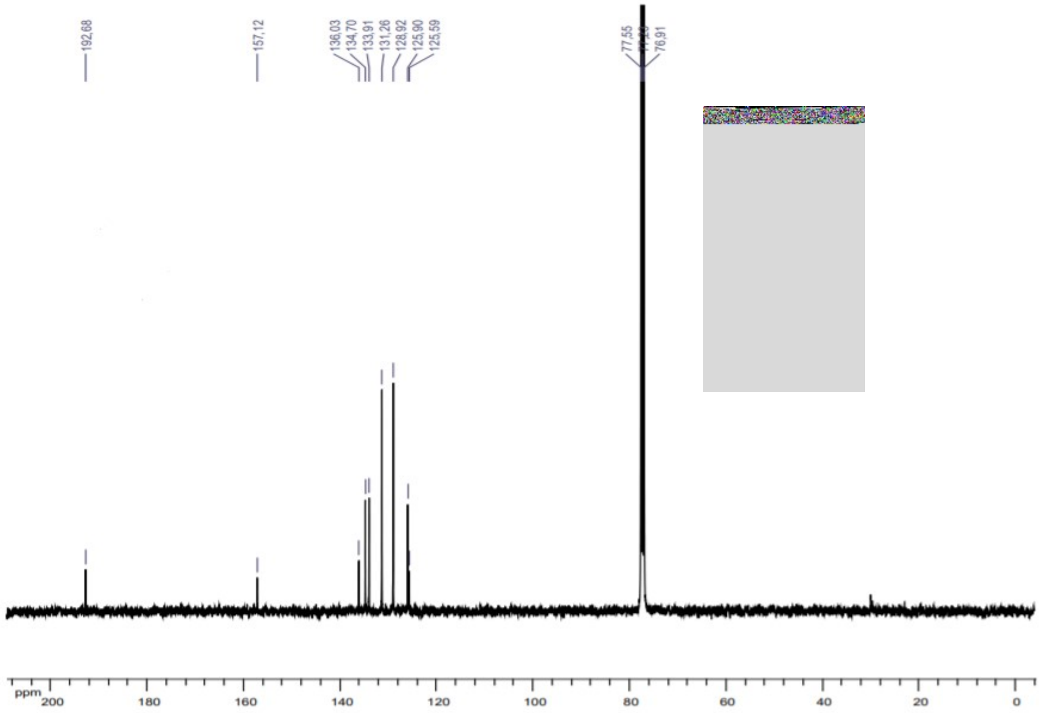

^{13}C NMR of Phthalazine-1,4-diylbis(phenyl)methanone **(8)**

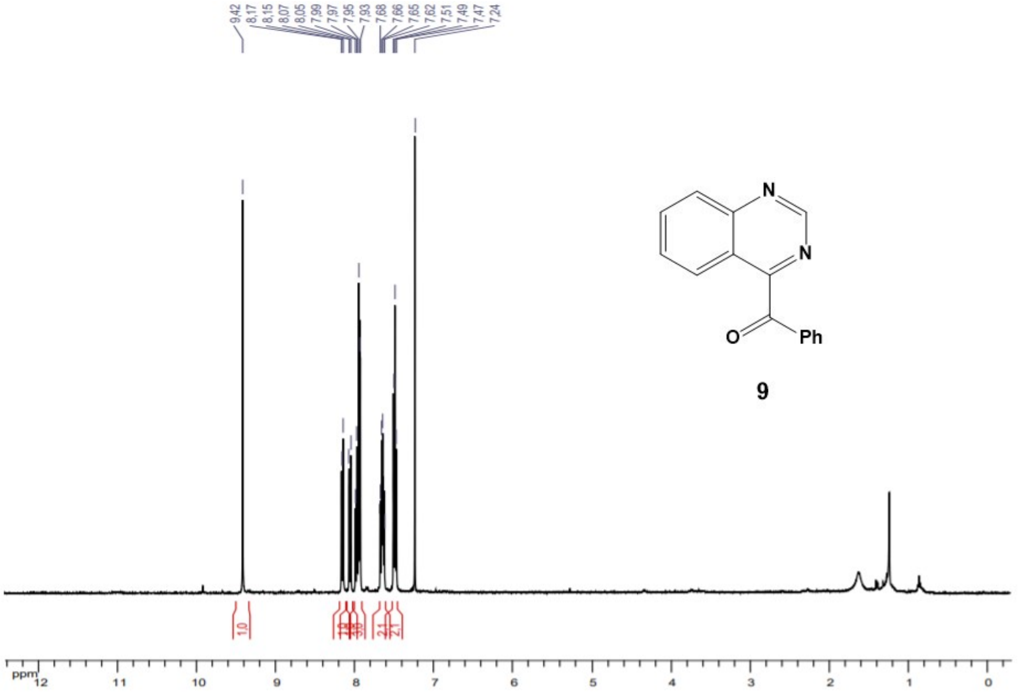

^{1}H NMR of 4-benzoylquinazoline (**9**)

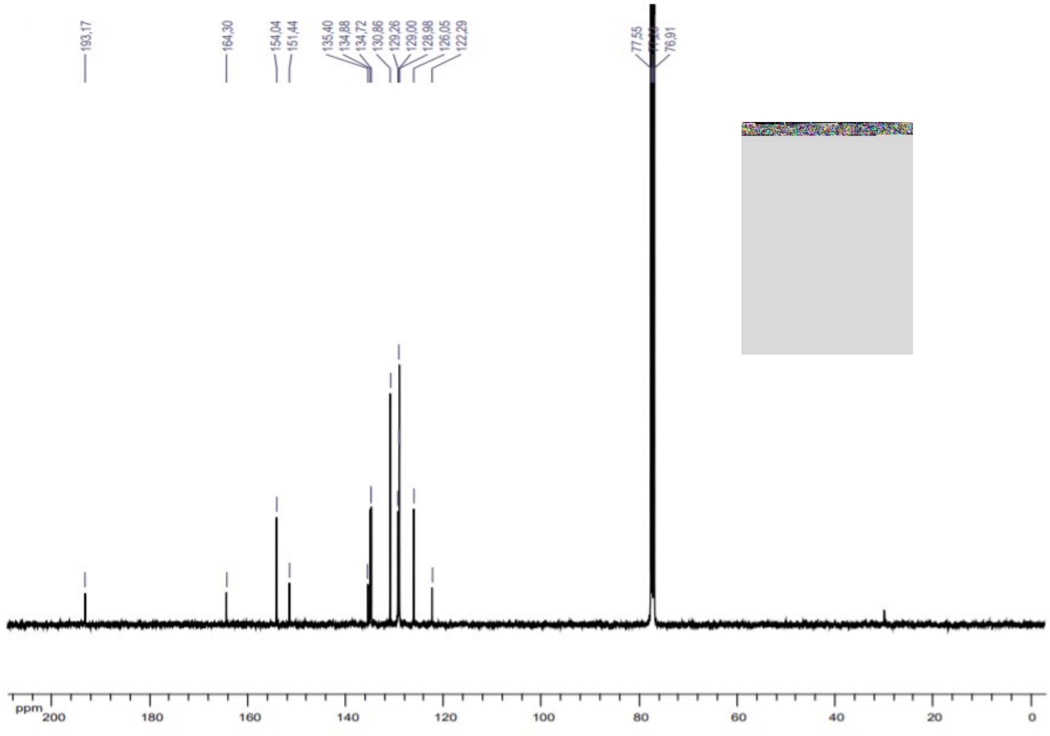

^{13}C NMR of 4-benzoylquinazoline (**9**)

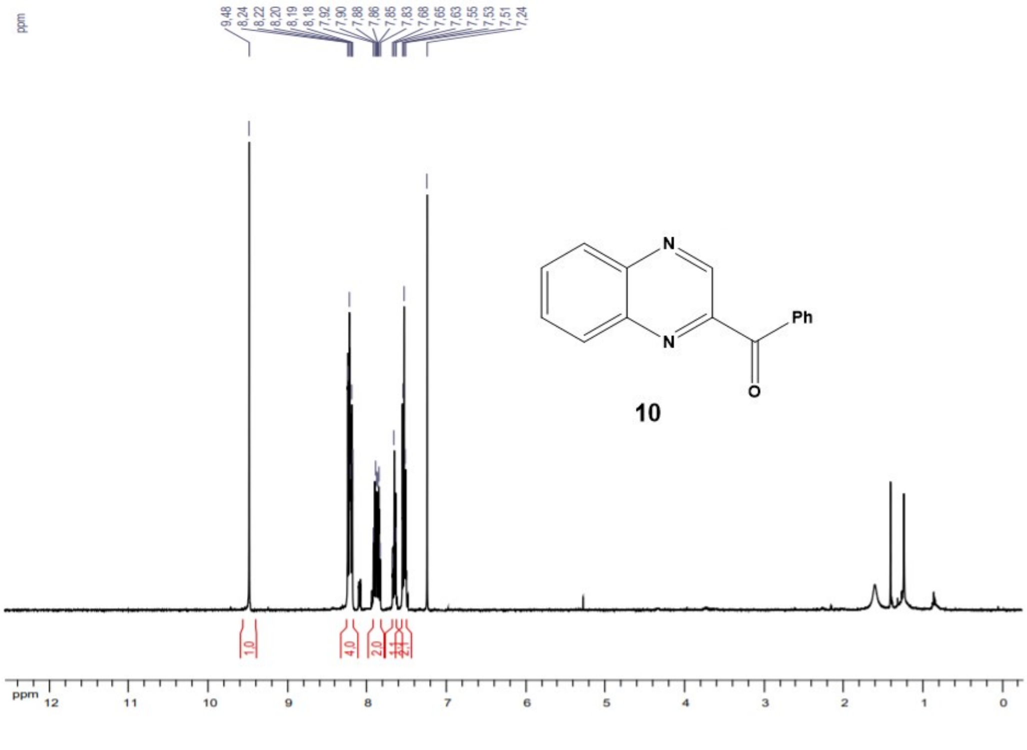

^{1}H NMR of Phenyl(quinoxalin-2-yl) methanone **(10)**

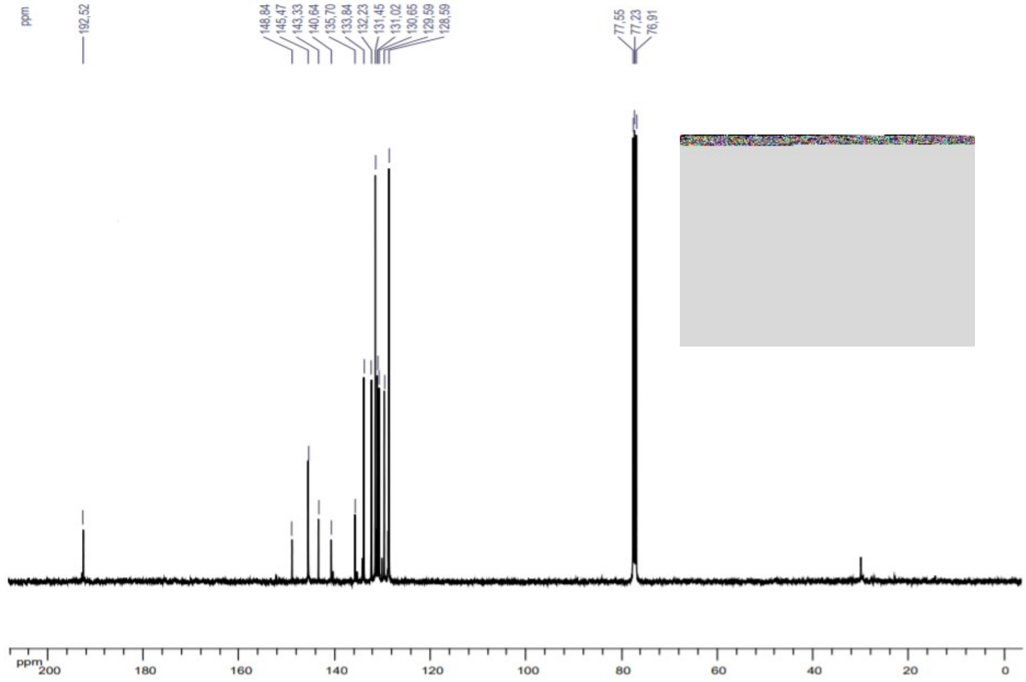

^{13}C NMR of Phenyl(quinoxalin-2-yl) methanone **(10)**

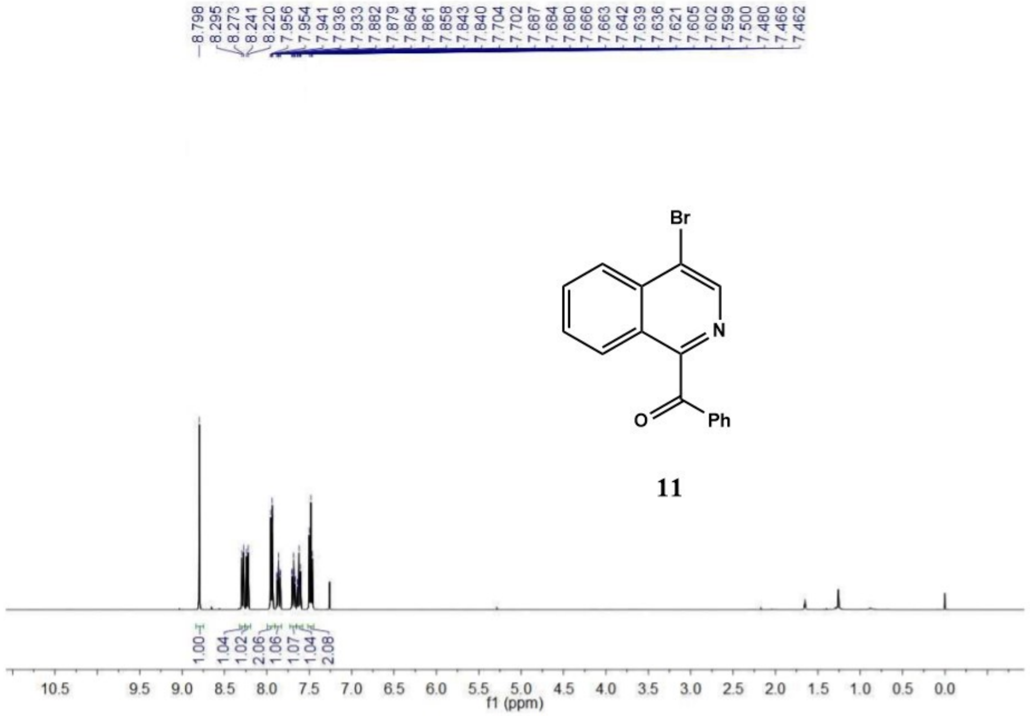

^{1}H NMR of (4-Bromoisoquinolin-1-yl) (phenyl)methanone **(11)**

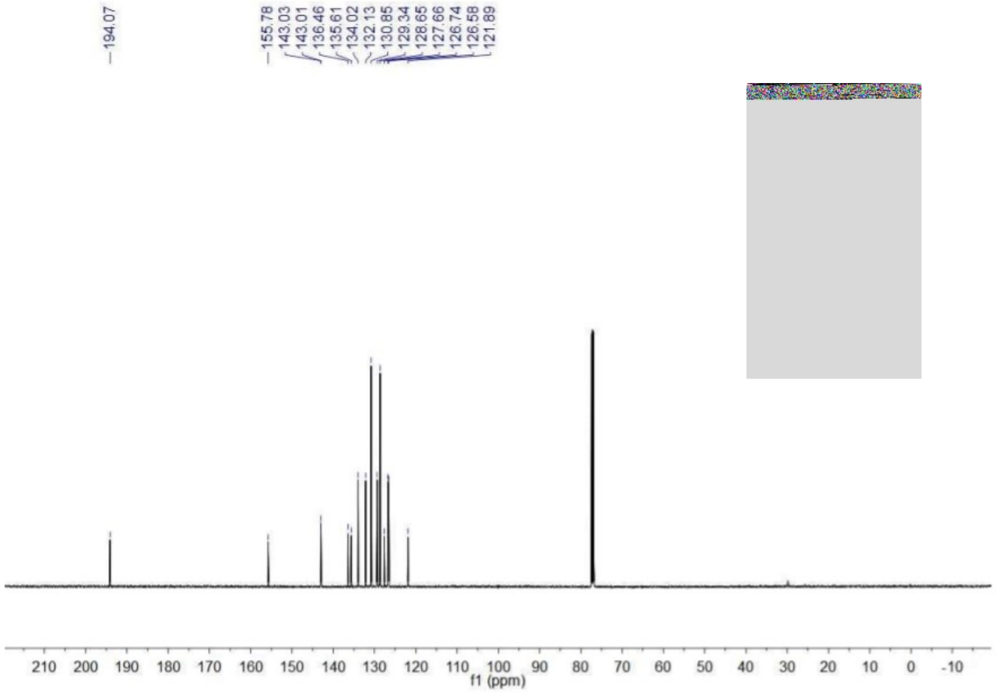

^{13}C NMR of (4-Bromoisoquinolin-1-yl) (phenyl)methanone **(11)**

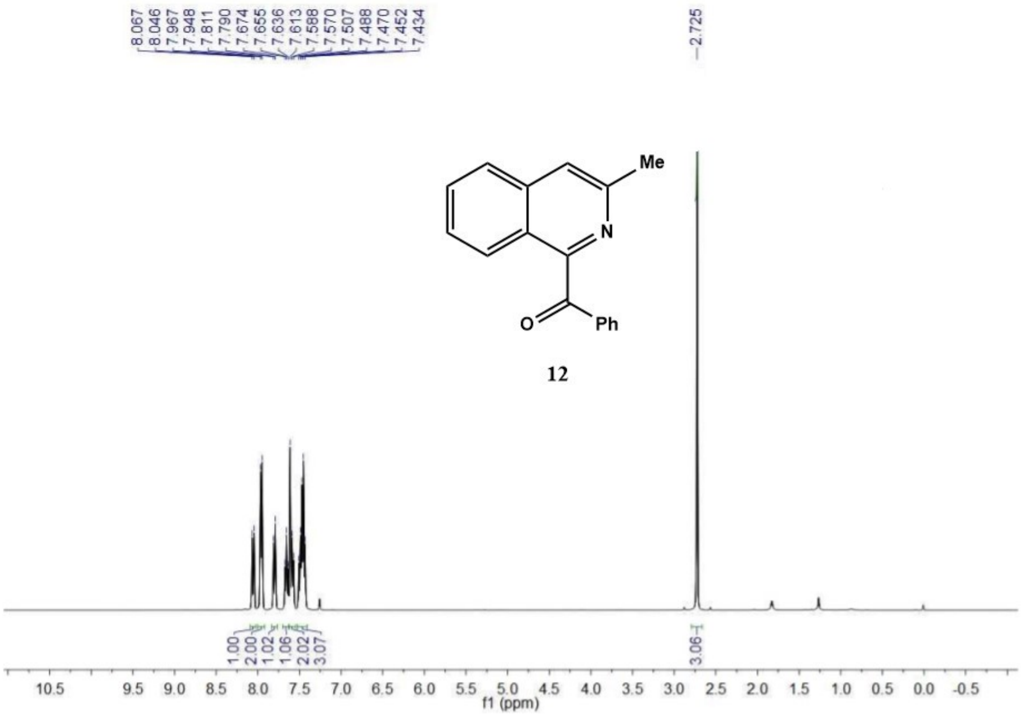

^{1}H NMR of (3-Methylisoquinolin-1-yl) (phenyl)methanone **(12)**

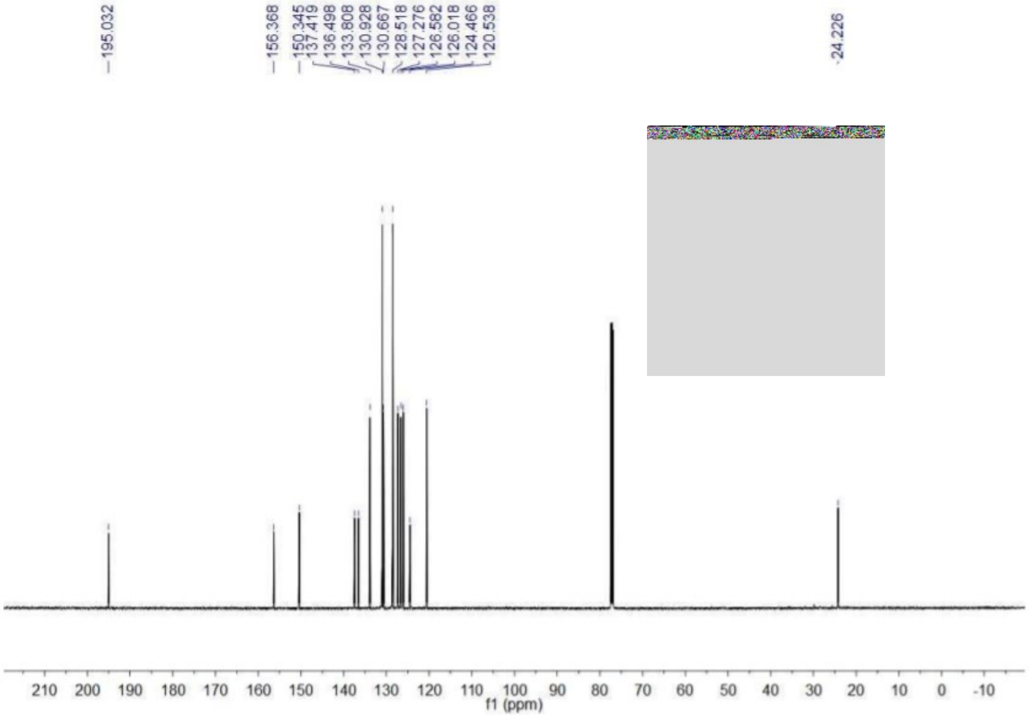

^{13}C NMR of (3-Methylisoquinolin-1-yl) (phenyl)methanone **(12)**

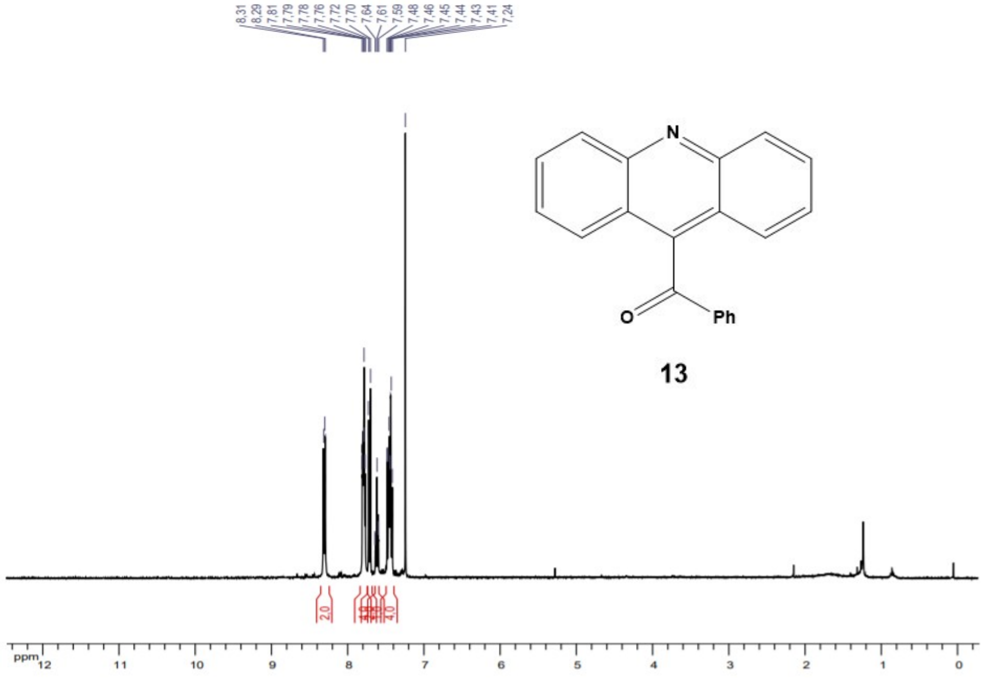

^{1}H NMR of Acridin-9-yl(phenyl)methanone **(13)**

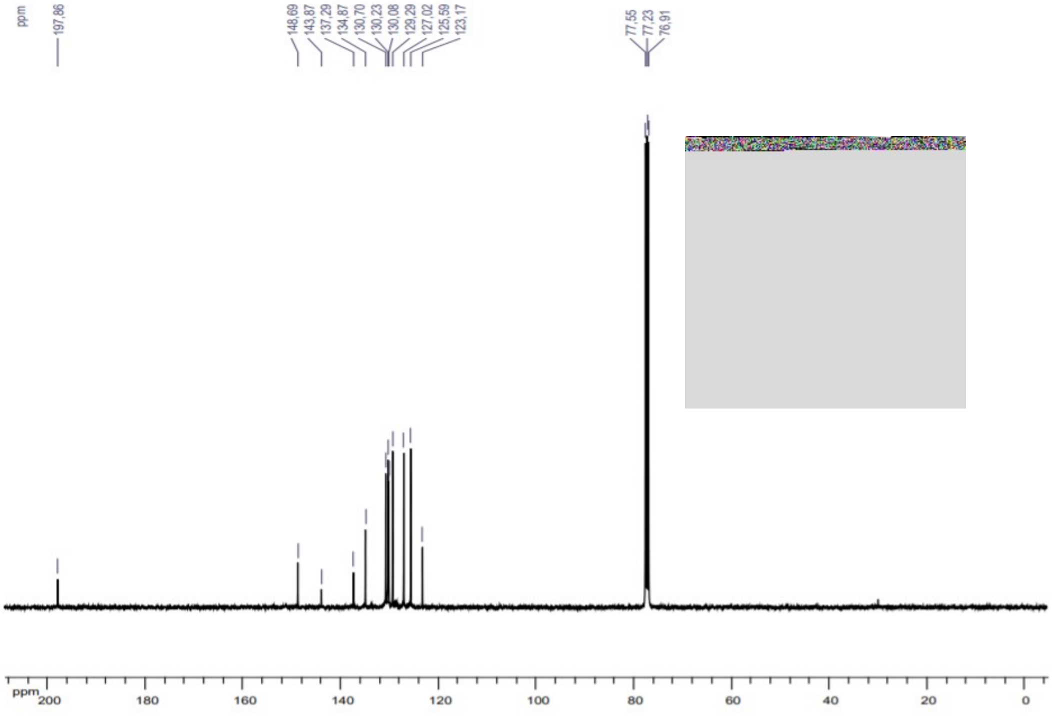

^{13}C NMR of Acridin-9-yl(phenyl)methanone **(13)**

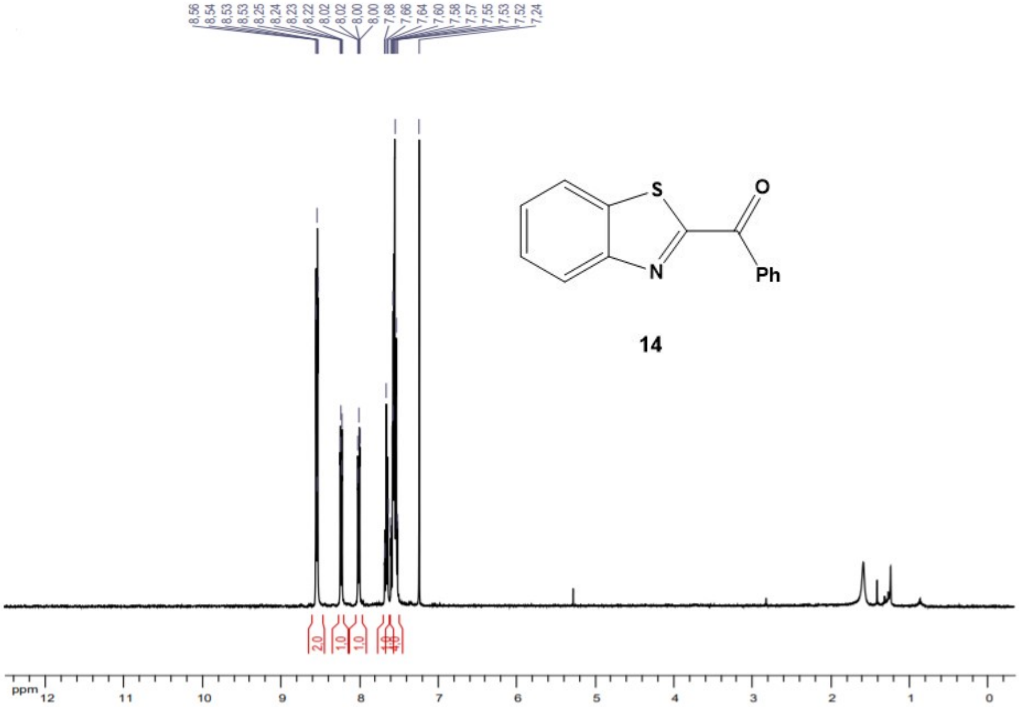

^{1}H NMR of Benzo[d]thiazol-2-yl(phenyl)methanone (**14**)

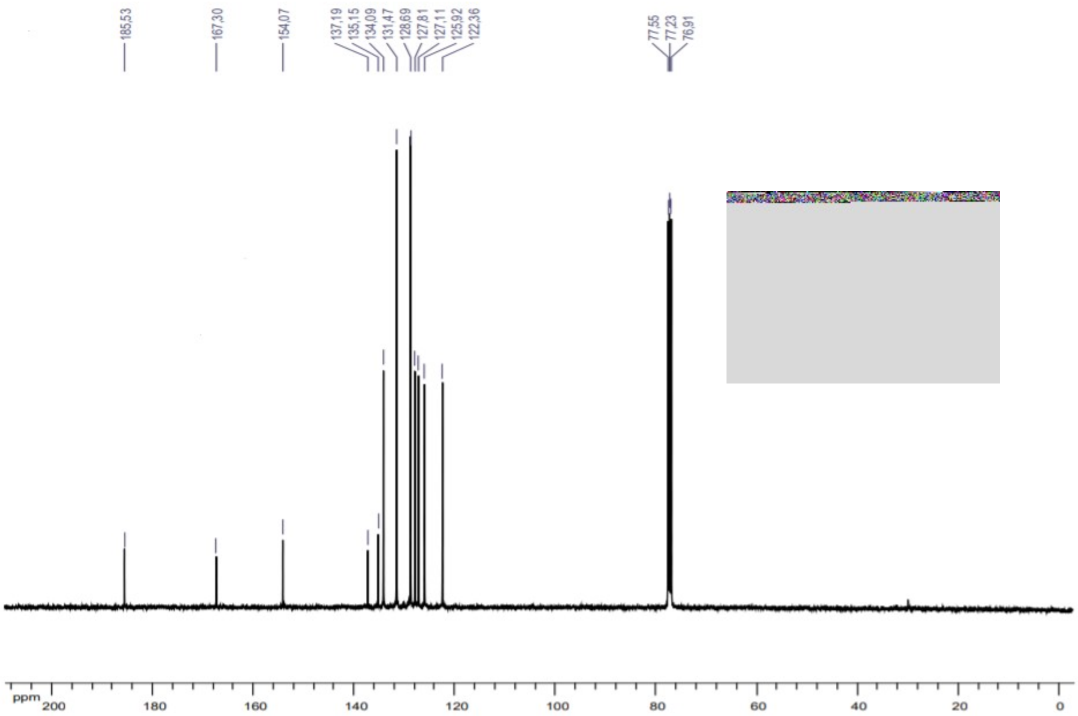

^{13}C NMR of Benzo[d]thiazol-2-yl(phenyl)methanone (**14**)

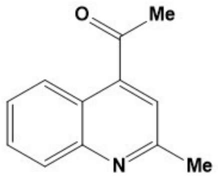

15

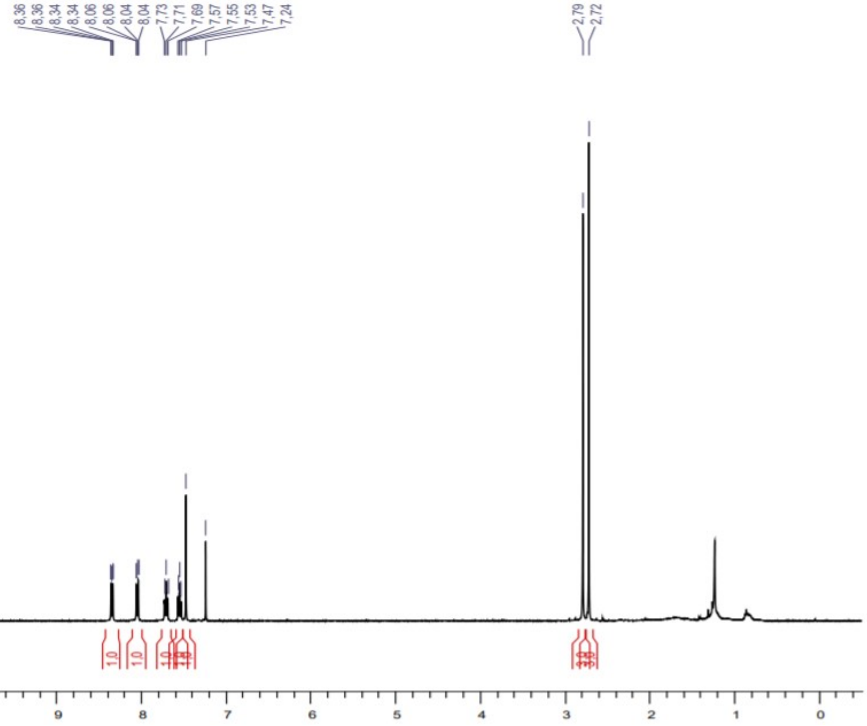

^{1}H NMR of 1-(2-methylquinolin-4-yl) ethane-1-one (**15**)

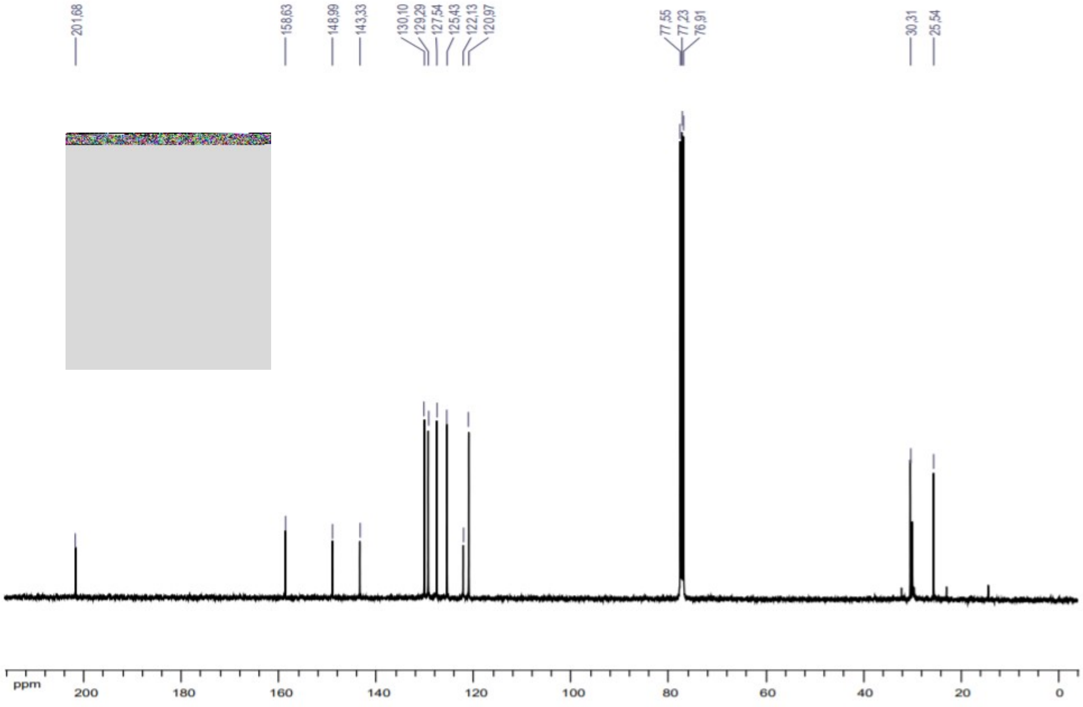

^{13}C NMR of 1-(2-methylquinolin-4-yl) ethane-1-one (**15**)

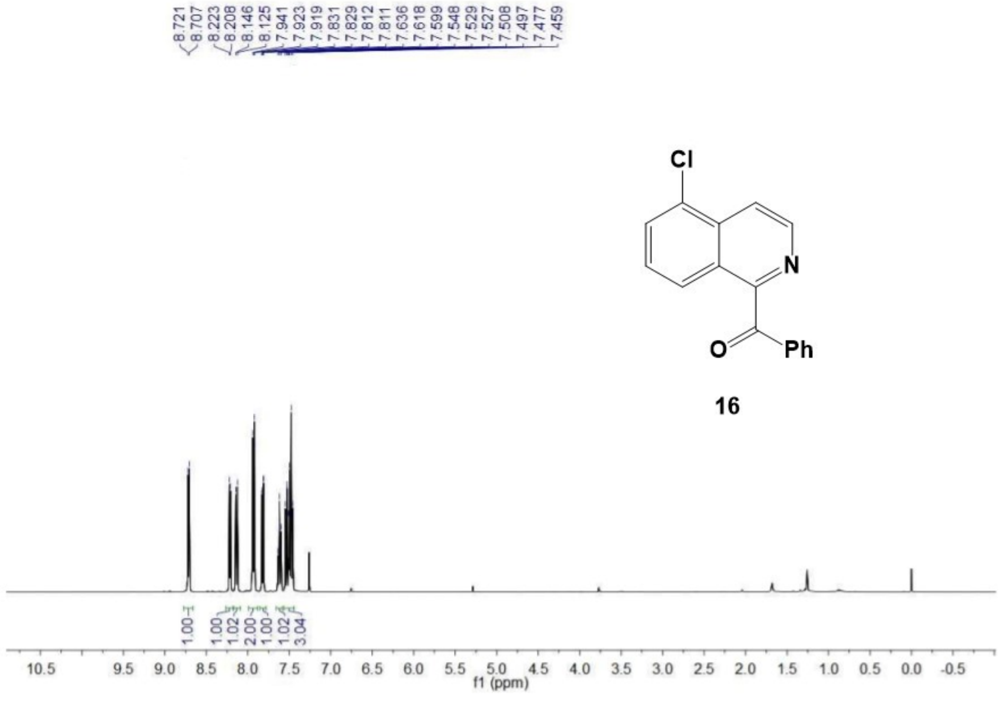

^{1}H NMR of (5-Chlorisoquinolin-1-yl) (phenyl)methanone **(16)**

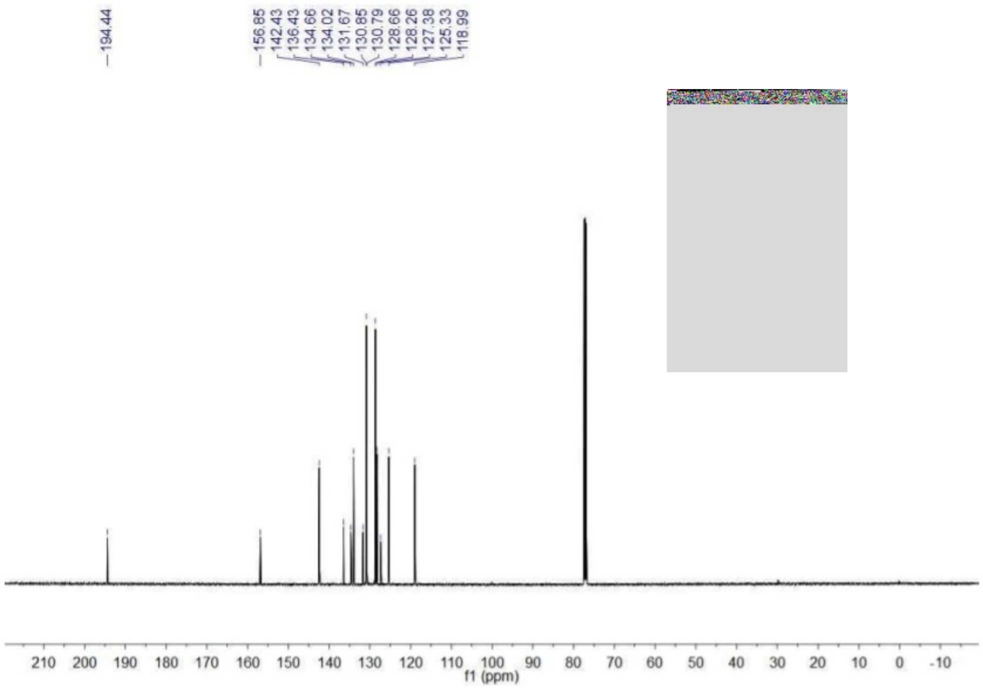

^{13}C NMR of (5-Chloroisoquinolin-1-yl) (phenyl)methanone **(16)**

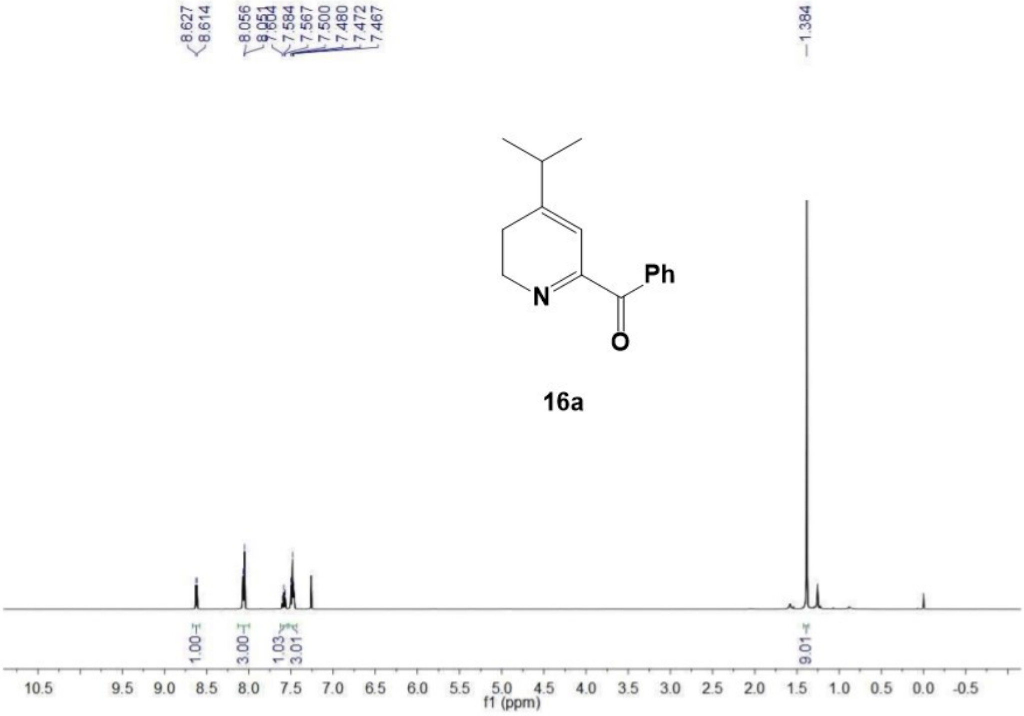

^{1}H NMR of (4-(tert-butyl)pyridin-2-yl)(phenyl)methanone (**16a**)

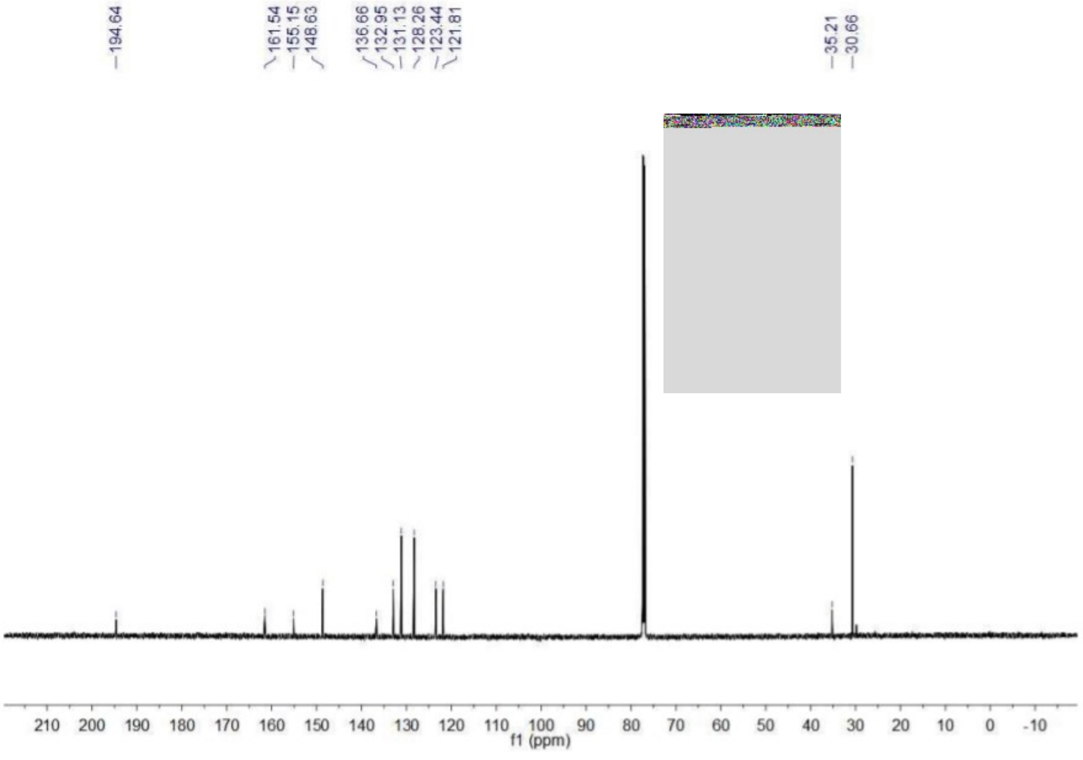

^{13}C NMR of (4-(tert-butyl)pyridin-2-yl)(phenyl)methanone (**16a**)

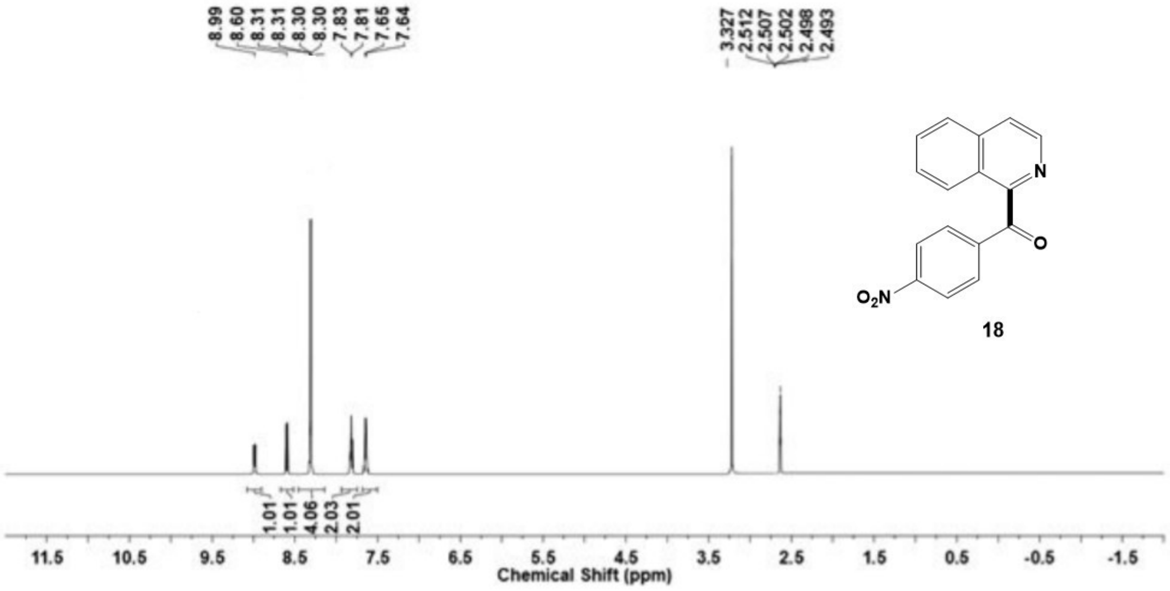

^{1}H NMR of Isoquinolinyl(4-nitrophenyl)methanone (**18**)

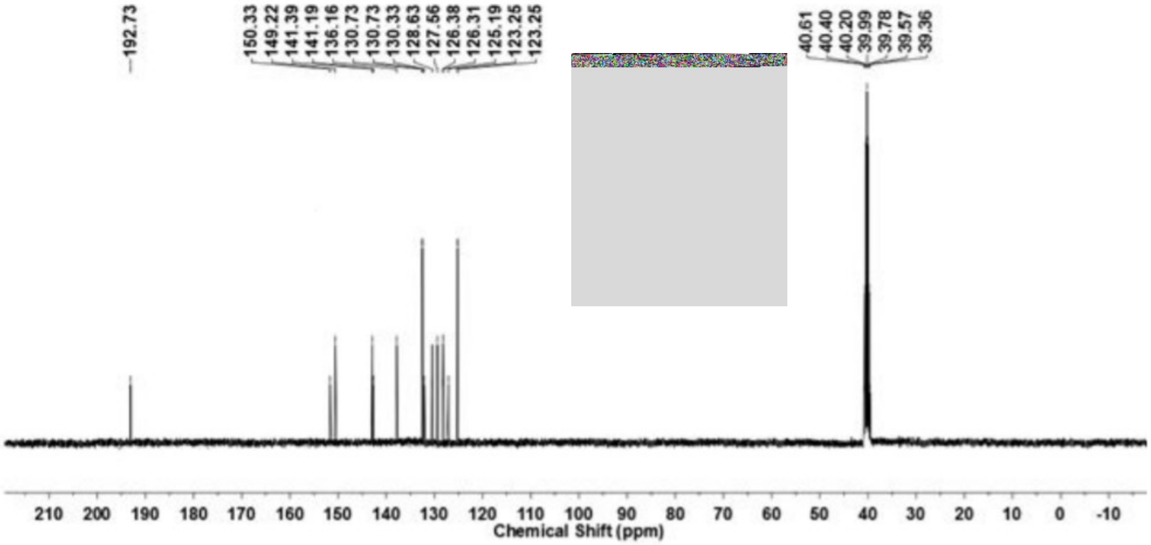

^{13}C NMR of Isoquinolinyl(4-nitrophenyl)methanone (**18**)

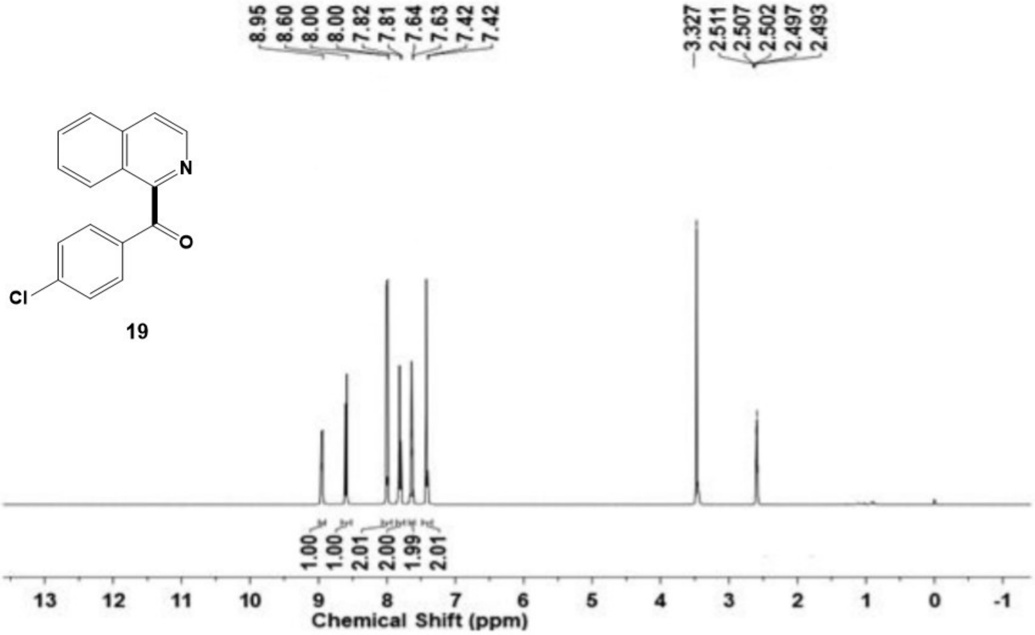

^{1}H NMR of Isoquinolinyl(4-chlorophenyl)methanone (**19**)

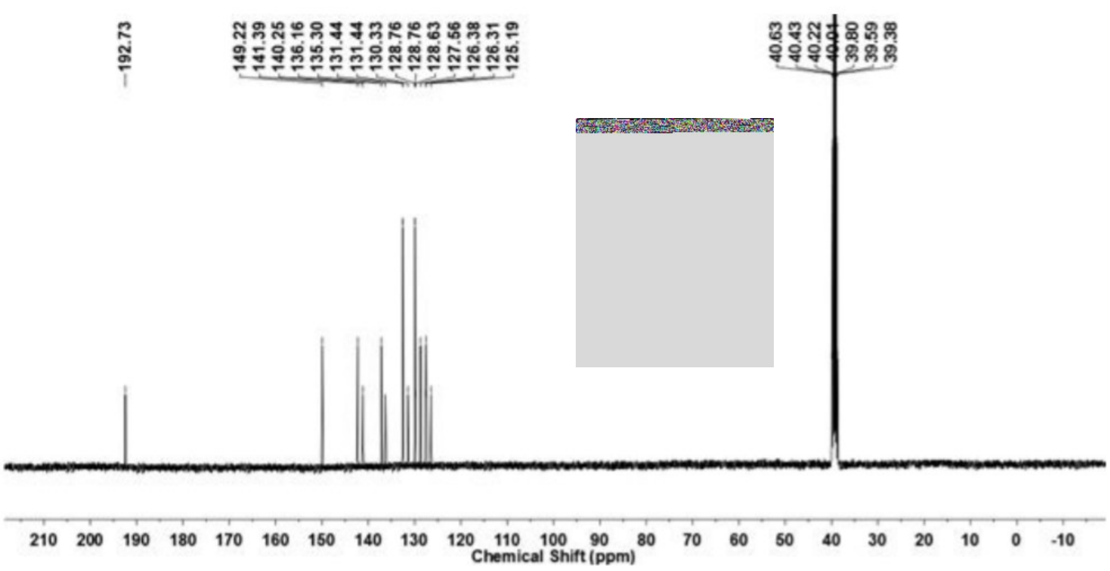

^{13}C NMR of Isoquinolinyl(4-chlorophenyl)methanone (**19)**
